# Genetic Pleiotropy between Nicotine Dependence and Respiratory Outcomes

**DOI:** 10.1038/s41598-017-16964-4

**Published:** 2017-12-04

**Authors:** Jushan Zhang, Shouneng Peng, Haoxiang Cheng, Yoko Nomura, Antonio Fabio Di Narzo, Ke Hao

**Affiliations:** 1 Department of Respiratory Medicine, Shanghai Tenth People’s Hospital, Tongji University, Shanghai 200072, China; 2 School of Life Sciences and Technology, Tongji University, Shanghai 200092, China; 3 Department of Genetics and Genomic Sciences, Icahn School of Medicine at Mount Sinai, New York, NY 10029 USA; 4 Icahn Institute of Genomics and Multiscale Biology, Icahn School of Medicine at Mount Sinai, New York, NY 10029 USA; 5 Department of Psychology, Queens College & Graduate Center, the City University of New York, New York, NY 10016 USA; 6 Department of Psychiatry, Icahn School of Medicine at Mount Sinai, New York, NY 10029 USA

## Abstract

Smoking is a major cause of respiratory conditions. To date, the genetic pleiotropy between smoking behavior and lung function/chronic obstructive pulmonary disease (COPD) have not been systematically explored. We leverage large data sets of smoking behavior, lung function and COPD, and addressed two questions, (1) whether the genetic predisposition of nicotine dependence influence COPD risk and lung function; and (2) the genetic pleiotropy follow causal or independent model. We found the genetic predisposition of nicotine dependence was associated with COPD risk, even after adjusting for smoking behavior, indicating genetic pleiotropy and independent model. Two known nicotine dependent loci (15q25.1 and 19q13.2) were associated with smoking adjusted lung function, and 15q25.1 reached genome-wide significance. At various suggestive p-value thresholds, the smoking adjusted lung function traits share association signals with cigarettes per day and former smoking, substantially greater than random chance. Empirical data showed the genetic pleiotropy between nicotine dependence and COPD or lung function. The basis of pleiotropic effect is rather complex, attributable to a large number of genetic variants, and many variants functions through independent model, where the pleiotropic variants directly affect lung function, not mediated by influencing subjects’ smoking behavior.

## Introduction

Chronic obstructive pulmonary disease (COPD) is the third cause of death in the US after cancers and cardiovascular diseases^1^ and is among the leading causes of hospitalization in industrialized countries^2,3^. It was recently estimated that the absolute number of COPD cases in developed countries will increase by more than 150% from 2010 to 2030^4^, yet there is no curative therapy for COPD^1,5^. A lack of understanding of the molecular mechanisms in the pathogenesis of COPD has hampered efforts to develop new biomarkers and effective therapies.

COPD has multiple risk factors and complex etiology, where cigarette smoking and genetic susceptibility are among the main risk factors^1,6^. Tobacco smoking accounted for about 5.1 million deaths globally in 2004, and it is observed recent increases in smoking prevalence in developing countries^7^. But only 20–25% of smokers develop clinically significant airflow obstruction^8^. Smoking behavior is partially genetically determined, and at least genome-wide significant associated loci were identified in European ancestry subjects^9,10^, where the strongest and most consistent association reported is at the 15q25.1 locus (CHRNA3-CHRNA5-CHRNB4 gene cluster). Genes associated with smoking quantity were enriched for cholinergic receptors, sensory perception of smell, and Retinoid Binding^11^.

In parallel, there is strong evidence that genetic variants (both common and rare) contribution to COPD and pulmonary function. Candidate gene and genome-wide association studies (GWAS) have identified genetic variants associated with COPD^12–16^. The latest GWAS was performed by the International COPD Genetics Consortium (ICGC)^17^ identified 22 COPD susceptibility loci at genome-wide significance. Exome sequencing study found rare variants on CHRNA3, CHRNA5, and CHRNB4 genes were associated with COPD^18^. Lung function (e.g. FEV_1_, FVC and FEV_1_/FVC ratio) are traits closely related to COPD, in fact, lung function parameters are often used in defining COPD cases^6^. Three key parameters are commonly used in characterizing lung function: expired volume in 1st second (FEV_1_), forced vital capacity (FVC) and FEV_1_/FVC. FVC is the volume of air that can forcibly be blown out after full inspiration^19^, and is the most basic maneuver in spirometry tests. FEV_1_ is the volume of air exhaled in the first second during forced exhalation after maximal inspiration^19^. FEV_1_/FVC is the ratio of these two values, where the ratio ≥ 80% is considered normal^19^. To date, 97 independent genetic loci were identified influencing lung function traits (FEV_1_, FVC and FEV_1_/FVC)^6^.

**Figure 1:**
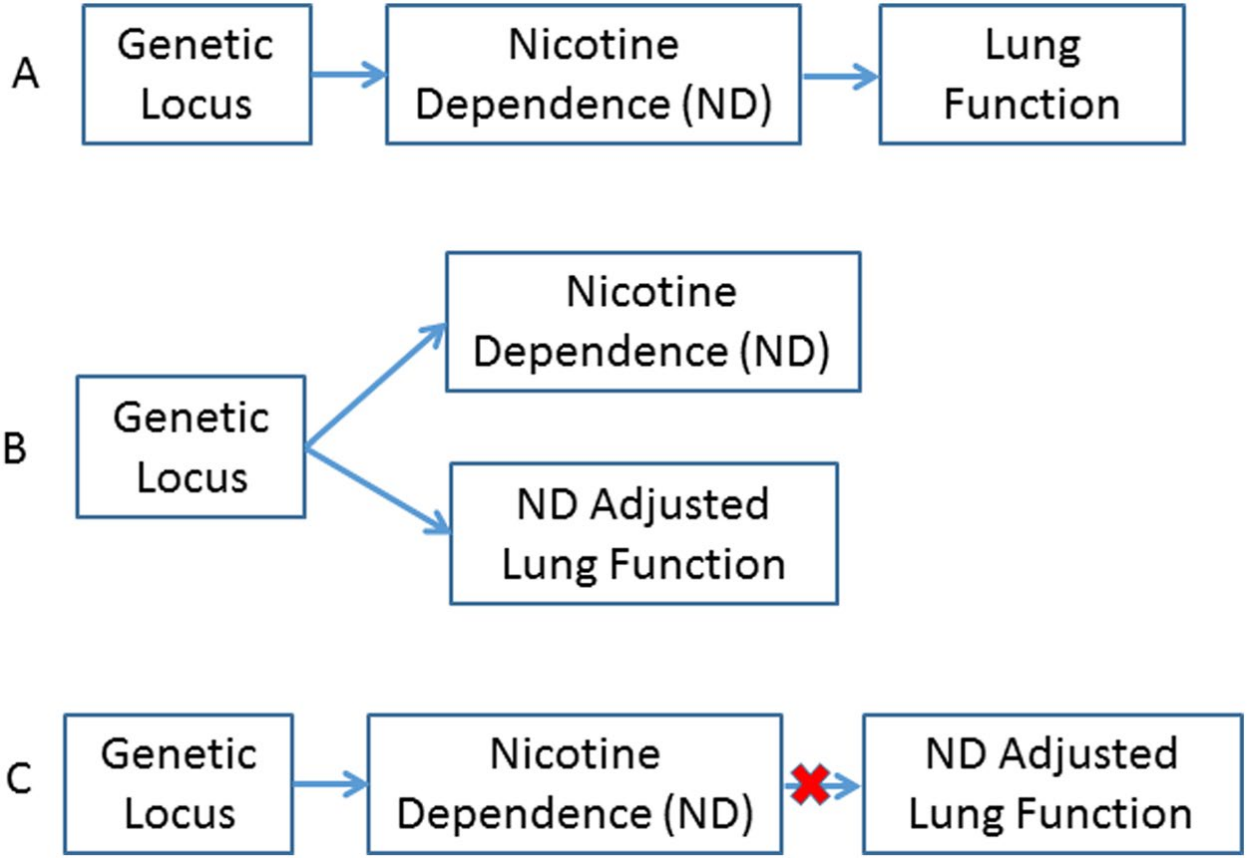
Models of genetic pleiotropy.

Genetic pleiotropy is the phenomenon where a DNA variant influences multiple traits^20^. Proposed by our group and others, available GWAS summary data can be used to detect genetic pleiotropy and pinpoint the variants, gene and pathways underlying the shared etiology^21–23^. Given smoking is a strong cause of COPD and lung function decline, genetic variants associated with smoking behavior or nicotine dependence (ND) are likely also in association with lung function (LF), which can be viewed as genetic pleiotropy. The genetic pleiotropy could have two possible mechanisms: (1) a genetic locus causes ND and then in turn causes LF decline (Fig. 1A); and (2) a genetic locus causes ND and LF decline independently (Fig. 1B). These two mechanisms can be distinguished by testing the association between genetic locus and smoking adjusted LF (ie, LF_adj_). If mechanism (1) is true, the ND locus will show no association with LF_adj_ (Fig. 1C).

**Figure 2:**
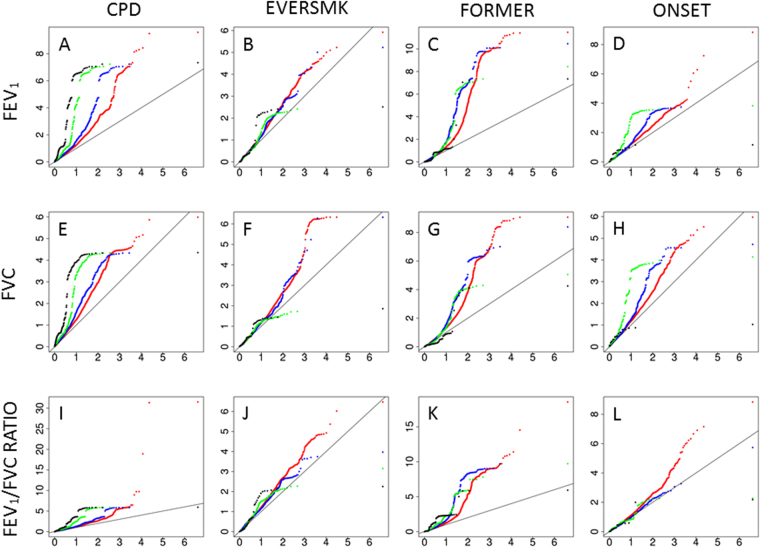
QQplot showed SNPs associated with nicotine dependence are enriched for small p values in lung function GWAS. The y axis of the plot of the top row (plot A, B, C and D) denotes the observed p value of smoking adjusted FEV_1_ in UKBB study; the y axis of the plot of the middle row (plot E, F, G and H) denotes the observed p value of smoking adjusted FVC in UKBB study; the y axis of the plot of the bottom row (plot I, J, K and L) denotes the observed p value of smoking adjusted FEV_1_/FVC Ratio in UKBB study. In the 1st column (plot A, E and I), we investigated on SNPs associated with cigarettes per day (CPD) at ≤1e-2 (red dots), ≤1e-3 (blue dots), ≤1e-4 (green dots), and ≤1e-5 (black dots); in the 2nd column (plot B, F and J), we investigated on SNPs associated with ever smoking (EVERSMK) at ≤1e-2 (red dots), ≤1e-3 (blue dots), ≤1e-4 (green dots), and ≤1e-5 (black dots); in the 3rd column (plot C, G and K), we investigated on SNPs associated with former smoking (FORMER) at ≤1e-2 (red dots), ≤1e-3 (blue dots), ≤1e-4 (green dots), and ≤1e-5 (black dots); in the 4th column (plot D, H and L), we investigated on SNPs associated with log of smoking onset (ONSET) at ≤1e-2 (red dots), ≤1e-3 (blue dots), ≤1e-4 (green dots), and ≤1e-5 (black dots).

A recent GWAS meta-analysis employed 48,943 UK Biobank individuals in discovery phase and 95,375 individuals in follow-up phase, and increased the independent signals for lung function from 54 to 97^6^. Importantly, the study carefully adjusted for smoking behavior, and among smokers, the pack-year was also adjustment^6^. The GWAS results of smoking-adjusted lung function offer a unique opportunity to decipher the genetic pleiotropy between nicotine dependence and function as well as the etiological mechanisms.

## Results

### Genetic predisposition of nicotine dependence is associated with COPD risk

We leverage the individual level phenotype (COPD affected status and smoking behavior) and genotype data of COPDgene cohort (Materials and Methods) to test association between genetic predisposition of nicotine dependence (quantitatively summarized as polygenic score, or PGS) and COPD risk. On each COPDgene study subject, we computed the PGS for ND CPD trait (i.e., PGS_ND-CDP_). The PGS_ND-CDP_ compiled at 1e-3 and 1e-4 p-value thresholds were associated with that COPD affected status at p-value 0.025 and 1.03e-6, respectively (Table 1), indicating significant pleiotropy of ND and COPD. The positive association coefficient indicate genetic predisposition of higher cigarettes per day leads to higher COPD risk (Table 1). PGS of former smoking (ie, PGS_ND-FORMER_) were also associated with COPD, and the negative coefficient suggested the genetic predisposition of smoking cessation reduced COPD risk. Next, we adjusted the smoking covariates and found the PGS_ND-CDP_ and PGS_ND-FORMER_ were still associated with COPD (Tables 2 and 3). Importantly, the association p value and coefficient were little changed before and after adjustment, indicating an independent pleiotropy model (Fig. 1B), where the effect of PGS_ND-CDP_ and PGS_ND-FORMER_ on COPD risk was not mediated by smoking behavior of the study participants.

**Table 1:** Polygenic score of nicotine dependence is associated with COPD case/control status

**PGS Training Trait**	**1e-3 p-value threshold in PGS construction**			**1e-4 p-value threshold in PGS construction**		
	^**#**^ **SNPs**	**β**	**p**.**value**	^**#**^ **SNPs**	**β**	**p**.**value**
Cigarettes Per Day	891	0.086	0.025495	116	0.190	1.03E-06
Ever Smoked	1110	0.082	0.030847	142	0.044	0.257046
Former Smoker	858	−0.138	2.37E-04	114	−0.069	0.078856
LogOnset	797	0.028	0.43318	76	−0.023	0.430121

Polygenic score (PGS) formulation is established based on TAG GWAS summary data using 1e-3 and 1e-4 p-value threshold on nicotine dependence (ND) traits. The ND PGS was computed on COPDgene dataset (sample size = 4903), and tested for association with COPD case-control status. ^#^SNPs, the number of SNPs used in PGS computation, β, association coefficient (ie, log Odds Ratio).

**Table 2:** Polygenic score of nicotine dependence is associated with COPD case/control status adjusted for smoking status

**PGS Training Trait**	**1e-3 p-value threshold in PGS construction**			**1e-4 p-value threshold in PGS construction**		
	^**#**^ **SNPs**	**β**	**p**.**value**	^**#**^ **SNPs**	**β**	**p**.**value**
Cigarettes Per Day	891	0.083	0.031482	116	0.185	2.07E-06
Ever Smoked	1110	0.090	0.017521	142	0.048	0.216163
Former Smoker	858	−0.143	1.49E-04	114	−0.066	0.091961
LogOnset	797	0.029	0.419497	76	−0.023	0.440345

Polygenic score (PGS) formulation is established based on TAG GWAS summary data using 1e-3 and 1e-4 p value threshold on nicotine dependence (ND) traits. The ND PGS was computed on COPDgene dataset (sample size = 4903), and tested for association with COPD case-control status, adjusted for smoking status (current smoker vs. non-smoker). ^#^SNPs, the number of SNPs used in PGS computation, β, association coefficient (ie, log Odds Ratio).

**Table 3:** Polygenic score of nicotine dependence is associated with COPD case/control status adjusted for smoking duration

**PGS Training Trait**	**1e-3 p-value threshold in PGS construction**			**1e-4 p-value threshold in PGS construction**		
	^**#**^ **SNPs**	**β**	**p**.**value**	^**#**^ **SNPs**	**β**	**p**.**value**
Cigarettes Per Day	891	0.085	0.0376	116	0.197	1.82E-06
Ever Smoked	1110	0.072	0.070447	142	0.057	0.163214
Former Smoker	858	−0.132	9.46E-04	114	−0.067	0.106736
LogOnset	797	0.022	0.555505	76	−0.019	0.544609

Polygenic score (PGS) formulation is established based on TAG GWAS summary data using 1e-3 and 1e-4 p value threshold on nicotine dependence (ND) traits. The ND PGS was computed on COPDgene dataset (sample size = 4903), and tested for association with COPD case-control status, adjusted for smoking duration. ^#^SNPs, the number of SNPs used in PGS computation, β, association coefficient (ie, log Odds Ratio).

### Nicotine dependence genome-wide significant loci and their association with lung function (LF)

Firstly, we retrieved six independent ND GWAS loci of genome-wide significance (p < 5e-8) in European ancestry^24^ from the NHGRI-EBI catalog^25^, and examined the association between these loci and smoking adjusted lung function (LF_adj_) in UKBB meta-analysis cohort (Table 4). In UKBB study, the smoking status was adjusted with the regression model in the form of indicator variables denoting current smoker, and denoting former smokers; as well as quantitative variable denoting pack-year (in smokers). At nominal p value (≤0.05), two ND loci showed association with at least one LF_adj_ traits (FEV_1_, FVC, and FEV_1_/FVC Ratio). The lead SNP, rs1051730, of the 15q25.1 locus was strongly associated with all three LF_adj_ trait, and its association with smoking adjusted FEV_1_ reached genome-wide significant (p = 5e-8), further, the 19q13.2 locus, known in association with COPD^26^, is also associated with smoking adjusted FEV_1_ (Table 4), suggesting the 15q25.1 and 19q13.2 loci could influence ND and LF independently (Fig. 1B). In contrast, four genome-wide significant ND loci (10q23.32, 8p11.21, 11p14.1, 9q34.2) showed no association with LF_adj_ traits (Table 4 and Fig S1), suggesting causal pleiotropy model (Fig. 1 A), where the genetic variants influence LF, mediated by smoking behavior.

**Table 4:** Nicotine dependence loci and in lung function.

Locus*	**Lead SNP**	**Chr**	**Position**	**Reported Genes**	**ND P**	**Association with LF**		
						**FEV**_**1**_	**FVC**	**RATIO**
15q25.1	rs1051730	15	78894339	CHRNA3	3.0E-73	**Y**	**Y**	**Y**
10q23.32	rs1329650	10	93348120	LOC100188947	6.0E-10	N	N	N
8p11.21	rs6474412	8	42550498	CHRNB3, CHRNA6	1.0E-08	N	N	N
19q13.2	rs3733829	19	41310571	EGLN2, CYP2A6, RAB4D	1.0E-08	**Y**	N	**Y**
11p14.1	rs6265	11	27679916	BDNF	2.0E-08	N	N	N
9q34.2	rs3025343	9	136478355	DBH	4.0E-08	N	N	N

^*^Genome-wide significant loci of nicotine dependence in European ancestry, 15q25.1^32^, 10q23.32^32^, 19q13.2^10,32^, 11p14.1^32^, 9q34.2^32^, 8p11.21^10^ were summarized by NHGRI-EBI GWAS catalog. The reported lead SNP was checked for association with smoking adjusted lung function in UKBB GWAS meta-analysis^6^. ND, nicotine dependence; LF, lung function.

### Identification of SNPs associated with lung function and nicotine dependence at suggestive threshold

There is a general consensus among GWAS studies that a p-value less than 5e-8 corresponds to genome-wide significance^24^. NHGRI-EBI GWAS catalog focuses on highly significant loci, but true association loci may only show suggestive p values given the limited power in GWAS. We leverage the summary data of with UKBB smoking adjusted LF (LF_adj_) and TAG ND (Materials and Methods). Applied various p thresholds (ranging from 1e-8 to 1e-2), we identified SNPs in associated with LF_adj_ or ND (Table S1). The UKBB LF_adj_ GWAS yield many SNPs highly significantly associated with smoking adjusted FEV_1_, FVC and FEV_1_/FVC Ratio. On the other hand, the TAG ND study captured strong genetic signals for CPD (cigarettes per day) and moderate signal for former smoking (Table S1). But no SNP associated ever smoking or log-onset at p < 1e-7 level, indicating these two traits either are not controlled by genetic factors or the TAG study did not have sufficient power (Table S1).

Among the ~2 million SNPs tested in both LF_adj_ and ND GWAS studies, the proportion of associated SNPs at any given p-value threshold was greater than alpha level (Table 5). For example, at p-value ≤ 1e-3 threshold, 11,716 SNPs and 2,791 SNPs were associated with FEV_1_ and CPD, respectively, and 71 SNPs were associated with both trait, which is substantially greater than random chance (enrichment fold = 4.61). In fact, we observed excess overlapping of FEV_1_ and CPD GWAS SNPs were across multiple p-value thresholds (Table 5 and Fig. 2). Further, at p value ≤ 1e-3 threshold, 139 SNPs were associated with both FEV_1_ and former smoking (Table 6), 11.8 folds of enrichment than random chance. While, GWAS signals of ever smoking and smoking onset showed little or very modest overlap with LF_adj_ GWAS (Tables S2 and S3). The SNPs associated with at least one of the nicotine dependence and lung function traits (cigarettes per day, former smoking, FEV_1_, FVC, FEV_1_/FVC) at p value ≤ 1e-3 level were listed in Table S4; while SNPs associated with all of the five nicotine dependence and lung function traits were detailed in Table S4.

**Table 5:** Overlap of GWAS signals between nicotine dependence (cigarettes per day) and lung function traits.

**Lung Function Traits**	**GWAS P value threshold**	**Enrichment Fold**	**N of overlap SNPs**	**N of overlap SNPs of divergent risk direction**	**% SNPs of divergent risk direction**	**p-value of divergent risk direction**
FEV_1_	1.0E-06	261	26	26	100	6.32E-08
	1.0E-05	127	26	26	100	6.32E-08
	1.0E-04	54.9	42	42	100	1.35E-12
	1.0E-03	4.61	71	65	91.55	4.34E-13
	1.0E-02	1.67	826	451	54.60	1.29E-2
	1.0E-01	1.03	29,067	15,404	52.99	9.00E-24
FVC	1.0E-06	—	0	0	—	—
	1.0E-05	—	0	0	—	—
	1.0E-04	19.1	13	13	100	3.91E-04
	1.0E-03	3.50	49	47	95.92	1.17E-11
	1.0E-02	1.81	869	491	56.50	2.40E-04
	1.0E-01	1.07	29,865	15,840	53.04	4.47E-25
FEV_1_/FVC Ratio	1.0E-06	—	0	0	—	—
	1.0E-05	63.90	14	14	100	2.08E-04
	1.0E-04	14.60	14	14	100	2.08E-04
	1.0E-03	2.28	39	39	100	9.80E-12
	1.0E-02	1.31	669	407	60.84	5.23E-08
	1.0E-01	1.03	28,468	14,474	50.84	6.70E-03

^*^Consistent direction allele, the SNPs where the same allele association with higher cigarettes per day and higher lung function trait (e.g. FEV_1_).

**Table 6:** Overlap of GWAS signals between nicotine dependence (former smoking) and lung function traits.

**Lung Function Traits**	**GWAS P value threshold**	**Enrichment Fold**	**N. overlap SNPs**	**N of overlap SNPs of divergent risk direction**	**% SNPs of divergent risk direction**	**p-value of divergent risk direction**
FEV_1_	1.0E-06	—	0	0	—	—
	1.0E-05	22.9	4	0	0	1.53E-01
	1.0E-04	17.1	20	0	0	3.59E-06
	1.0E-03	11.8	193	21	10.88	6.69E-30
	1.0E-02	2.29	1202	290	24.13	5.74E-74
	1.0E-01	1.07	30328	13490	44.48	4.54E-81
FVC	1.0E-06	—	0	0	—	—
	1.0E-05	—	0	0	—	—
	1.0E-04	8.24	8	0	0	1.13E-02
	1.0E-03	9.68	144	21	14.58	3.94E-18
	1.0E-02	2.14	1099	336	30.57	6.23E-38
	1.0E-01	1.06	30050	13453	44.77	2.26E-72
FEV_1_/FVC Ratio	1.0E-06	—	0	0	—	—
	1.0E-05	23.5	4	0	0	1.53E-01
	1.0E-04	17.3	23	0	0	4.77E-07
	1.0E-03	9.35	168	3	1.79	3.49E-44
	1.0E-02	1.98	1066	343	32.18	5.01E-31
	1.0E-01	1.04	28980	13923	48.04	7.26E-11

^*^Consistent direction allele, the SNPs where the same allele association with higher odds of former smoking (ie, smoking cessation) and higher lung function trait (e.g FEV_1_).

### Association direction of shared LF_adj_ and ND SNPs

The shared LF_adj_/ND SNPs can be stratified into categories according whether the allele associated with higher LF_adj_ trait is also associated with higher ND trait or ND event odds (ie, consistent direction SNP) or vice versa (ie, divergent direction SNP). We stratified the shared LF_adj_/ND SNPs by directions, and found all three LF_adj_ traits primarily have divergent direction with CDP (Table 5). At 1e-3 threshold, 71 SNPs are shared by FEV_1_ and CPD, where 65 SNPs (91.55%) with divergent directions (binomial p value = 4.34e-13), indicating alleles lead to higher smoking quantity (cigarettes per day) tends to associated with lower LF_adj_ traits. In contrast, the LF_adj_ traits primarily have consistent direction with former smoking (Table 6). At 1e-3 threshold, 193 SNPs were shared by FEV_1_ and former smoking, and only 21 SNPs (10.88%) with divergent directions but 172 SNPs are of a consistent direction (binomial p-value = 4.34e-13), indicating alleles lead to smoking cessation tend to associated with higher LF_adj_ traits.

Further, we stratified the SNPs into the consistent direction SNP and divergent SNP subcategories, regardless of GWAS test p-values criteria (Tables S5–S8). We found the overlap of CPD and LF_adj_ GWAS signals primarily occur among the SNPs of divergent allele direction, and observed little overlap among the SNPs of consistent allele direction. For example, at a GWAS p-value < 1e-3, SNPs of divergent allele direction for FEV_1_ and CPD showed substantial overlap (6.94 fold enrichment), in contrast, the overlap for SNPs of consistent allele direction were not different from random chance.

### Gene ontology influenced by the shared SNPs of LF_adj_ and ND

**Figure 3:**
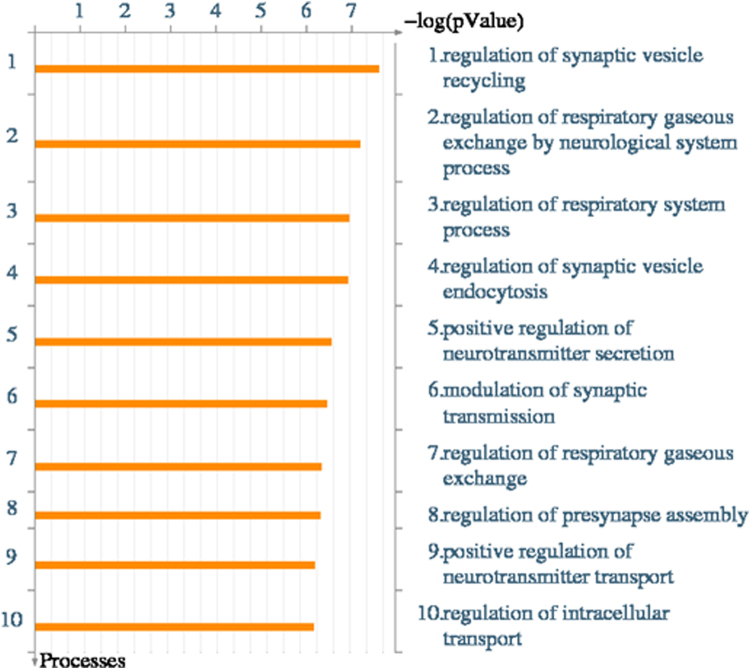
Genes harbor or close to share SNPs between cigarettes per day and smoking adjusted FEV_1_ are enriched for certain Gene Ontology cellular processes.

Shared LF_adj_/ND SNPs may undergo complicated pathways and lead to disease risk. Identifying the genes influenced by such shared SNPs would be the key step elucidating the SNPs’ function and pathogenic pathways. To investigate potential functional impacts of LF_adj_/ND shared SNPs, we used a “relax” p-value threshold criterion of 1e-2 for GWAS. Among the 826 shared FEV_1_ - CPD SNPs, 375 are characterized by having consistent direction and 451 are characterized by divergent direction. Based on SNP position and dbSNP annotation^27^, we identified genes located with on or near the share GWAS SNPs of LF_adj_ and ND, and then carried out the Gene Ontology (GO) cellular processes enrichment analysis using METACORE suite (Fig. 3). The top 10 enriched processes (p-value ≤ 1e-6) were regulation of synapse vesicles as well as regulation of respiratory systems, highly relevant to lung function and nicotine dependence.

### Co-localization of 15q25.1 locus underlying LF_adj_ and ND

SNPs on 15q25.1 locus are associated with both LF_adj_ and ND at genome-wide significant level (Table 4). Also, the CHRNA3-CHRNA5-CHRNB4 gene cluster in this locus is functional relevant to both traits. However, given that neighboring SNPs were often in tight linkage disequilibrium (LD), the overlap of GWAS signals do not guarantee that the disease risks of the two traits are caused by the same variant. Recently developed methods allow more advanced integration of GWAS summary data to co-localize GWAS signals^28^. The co-localization methods^28^ evaluated 5 hypotheses (Materials and Methods), where we were particularly interested in hypothesis 4 (H4: the two phenotypic traits were caused by the same SNP in the locus), and H4 posterior probability over 0.75 was considered as supporting evidence to the corresponding hypothesis^29,30^. All the three LF_adj_ trait (FEV_1_, FVC and FEV_1_/FVC Ratio) were co-localized at 15q25.1 (Table 7), indicating they are controlled by the same genetic variant. The cigarettes per day and former smoking traits were also co-localized with LF_adj_ (Table 8). For example, between CPD and FEV_1_, the coloc H_4_ posterior probability was 0.908, and between former smoking and FEV_1_, the coloc H_4_ posterior probability was 0.942. The clear co-localization of LF_adj_ and ND at 15q25.1 suggested same genetic variant influence both nicotine dependence and lung function, through an independent pleiotropy model.

**Table 7:** Co-localization of lung function GWAS signal at 15q25.1 locus*.

**Trait 1**	**Trait 2**	**N SNPs**	**PP**.**H0**	**PP**.**H1**	**PP**.**H2**	**PP**.**H3**	**PP**.**H4**
FEV_1_	FVC	13040	0.000149	0.0653	0.000266	0.115461	0.818824
FEV_1_	FEV_1_/FVC Ratio	13041	4.85E-06	0.002121	9.77E-05	0.041758	0.956019
FVC	FEV_1_/FVC Ratio	13040	0.004064	0.007236	0.081852	0.144994	0.761854

^*^chr15:77,801,394-79,801,394 (HG19). Five COLOC hypothesis (H0~H4) were evaluated (Materials and Methods).

**Table 8:** Co-localization of lung function and nicotine dependence GWAS signal at 15q25.1 locus^*^.

**Trait 1**	**Trait 2**	**N SNPs**	**PP**.**H0**	**PP**.**H1**	**PP**.**H2**	**PP**.**H3**	**PP**.**H4**
FEV_1_	Cigarettes Per Day	1764	8.56E-32	1.46E-29	0.000537	0.091025	0.908438
	Ever Smoked	1762	0.005381	0.920954	0.000401	0.068663	0.004601
	Former Smoker	1758	9.76E-05	0.016708	0.000502	0.084986	0.897706
	LogOnset	1760	0.005601	0.95857	0.000172	0.029406	0.006252
FVC	Cigarettes Per Day	1764	2.52E-29	1.24E-29	0.157898	0.077069	0.765033
	Ever Smoked	1762	0.622132	0.306675	0.046387	0.022864	0.001942
	Former Smoker	1758	0.048471	0.023889	0.249148	0.122238	0.556253
	LogOnset	1760	0.648552	0.319665	0.0199	0.009806	0.002077
FEV_1_/FVC Ratio	Cigarettes Per Day	1764	1.17E-30	8.20E-30	0.007371	0.050554	0.942076
	Ever Smoked	1762	0.116032	0.810657	0.008652	0.060439	0.00422
	Former Smoker	1758	0.002055	0.01436	0.010565	0.072912	0.900107
	LogOnset	1760	0.120759	0.843642	0.003705	0.02588	0.006014

^*^chr15:77,801,394-79,801,394 (HG19). Five COLOC hypothesis (H0~H4) were evaluated (Materials and Methods).

## Discussion

In this report, we leverage the latest large GWAS data sets and investigated the genetic pleotropic effect between nicotine dependence and respiratory outcomes (ie, lung function and COPD). It is known smoking is a major cause of reduced lung function and COPD^8^. Smoking behavior is at least partially controlled by genetic factors^7,9^. To date, the pleiotropic effects of genetic risk of smoking behavior and lung function/COPD have not been systematically explored.

Employing several analytical approaches, this paper addressed two questions, (1) whether the genetic predisposition of nicotine dependence influence COPD risk and lung function; and (2) the genetic pleiotropy follow causal or independent model (Fig. 1). On COPDgene cohort, we found the polygenic score of nicotine dependence (ie, PGS_ND_) was associated with COPD case/control status, demonstrating the genetic pleiotropy of the two conditions. We also investigated the association between PGS_ND_ and COPD while adjusting smoking behavior. Interestingly, the crude and adjusted results were very similar, indicating a mainly independent pleiotropy model. That is the shared genetic factors directly modify COPD risk, not mediated by influencing the individual’s smoking behavior.

We zoomed in the known ND loci of genome-wide significance (Table 4), and found both causal and independent pleiotropy models may exist in certain loci. Two genome-wide significant ND loci (15q25.1 and 19q13.2) were associated with LF_adj_ (smoking adjusted lung function), supporting independent pleiotropy model. While, four ND loci (10q23.32, 8p11.21, 11p14.1 and 9q34.2) were not associated with smoking adjusted lung function. At various p value threshold (1e-6 to 1e-1), we found the LF_adj_ traits share association SNP with cigarettes per day and former smoking substantially more than random chance, indicating a large number of genetic variants contribute to the genetic pleiotropy. Importantly, the lung function and cigarettes per day mainly share SNPs of divergent direction, meaning genetic predisposition of higher smoking dosage leads to lower lung function. In contrast, the lung function and former mainly share SNPs of consistent direction, meaning genetic predisposition of smoking cessation leads to higher lung function.

In summary, we used empirical data of largest cohorts to date and showed the genetic pleiotropy between nicotine dependence and COPD or lung function. The pleiotropic effect exist even COPD status or lung function is adjusted for smoking behavior. Further, we found the pleiotropic effect is attributable not only to the genome-wide significant loci, but also loci associated to ND and COPD/LF at suggestive p value (e.g. 1e-3), suggesting a large number of variants influence both ND and respiratory outcome, and among which many variants functions through independent genetic pleiotropy model.

## Materials and Methods

### Genome-wide meta-analyses on nicotine dependence (ND)

The Tobacco and Genetics (TAG) Consortium conducted GWAS meta-analyses across 16 studies^10^. We examined four carefully harmonized smoking phenotypes: smoking initiation (ever versus never been a regular smoker), age of smoking initiation, smoking quantity (number of cigarettes smoked per day, CPD) and smoking cessation (former versus current smokers) among people of European ancestry. Smoking cessation contrasted former versus current smokers, where current smokers reported at interview that they presently smoked and former smokers had quit smoking at least 1 year before interview. Smokers who had quit smoking for less than 1 year at interview were excluded from the analysis to minimize misclassification. Genotype imputation resulted in a common set of ~2.5 million SNPs that entered the GWAS and meta-analysis, where summary statistics were used in this report.

### COPDGene dataset

Individual level genotype and phenotype data of COPDgene study were retrieved from dbGap (accession: phs000179.v1.p1). COPD case/control status, indicator variable of current smoking, indicator variable of former smoking, quantitative variable of smoking duration, and SNP genotype data were available on 4,903 individuals. We conducted genotype imputation using HRC reference^31^, and in total, 19,932,879 SNPs of high quality score entered the current study. In polygenic score analysis, the smoking status variable (indicator and quantitative variables) were adjusted within the regression model.

### UK Biobank Lung Function (LF) GWAS

Recently we reported a large GWAS study on lung function using the UK biobank samples^6^. Genome-wide association analyses of forced expired volume in 1 s (FEV_1_), forced vital capacity (FVC) and FEV_1_/FVC were undertaken in 48,943 individuals from the UK BiLEVE study^7^ who were selected from the extremes of the lung function distribution in UK Biobank (total n = 502,682). Association tests were conducted on 27,624,732 variants, where linear regression of age, age^2^, sex, height, the first ten principal components of genetic ancestry and pack-years of smoking (in smokers), and summary statistics were used in this report.

### Polygenic Score

We analyzed GWAS summary statistics data from the TAG nicotine dependence study, COPDgene dataset case control status, and COPDgene dataset smoking related covariate: smoking status (binary variable) and smoking duration (continuous variable). We computed the nicotine dependence polygenic score (PGS) on each COPDgene study subject in following steps: (1) identify shared SNPs in TAG GWAS summary data and COPDgene imputed genotype; (2) align alleles strands to the 1000 G panel (hg19), and adjust β coefficients TAG nicotine dependence GWAS accordingly; (3) filter TAG GWAS data by p value threshold (1e-3 and 1e-4 as shown in Table 5) and prune the SNPs by linkage disequilibrium (LD) based on 1000 G EUR reference; lastly (4) for each ND traits, and for each p value threshold, we computed a PGS for every subject as a linear combination of the imputed doses of the selected coefficients. Then we tested the association between nicotine dependent PGS and COPD case/control status using a logistic regression model with or without adjusting for smoking covariates.

### Identification of SNPs associated with both LF and ND

The effect size attributable to individual genetic variants for a given complex disorder is typically modest, suggesting that individual genetic variants may only explain a very small amount of the genetic risk and heritability of complex disorders^21,31^. Therefore, genetic contributions to complex conditions such as LF and ND are likely derived from a large number of genetic causal variants, each contributing a small genetic risk. We surveyed a number of p value thresholds, and identified SNPs that are associated with LF_adj_ and ND in order to more comprehensively capture SNPs with modest effect sizes. For a shared SNP, we term it “consistent allele direction” if a specific allele that is associated with increased LF_adj_ traits and that specific SNP allele is also associated with higher ND trait value or event odds. We term “divergent allele direction” for SNP where a specific allele associated with increased lung function, and lower ND trait value or ND event odds.

### Pathway Enrichment Analyses

To further characterize the regulatory nature, enrichment analysis of the genes influenced by shared SNPs were performed using the METACORE integrated software suite (http://thomsonreuters.com/metacore/).

### Co-localization of lung function and nicotine dependence GWAS top SNPs

LF_adj_ and ND GWAS results were used in co-localization analysis, which is performed using COLOC version 2.3–6 in R^28^. Our analysis focuses on the 15q25.1 locus. Default priors of the software were used. In total, 5 hypotheses were evaluated. H0: No association with either trait 1 or trait 2; H1: Association with trait 1, not with trait 2; H2: Association with trait 2, not with trait 1; H3: Association with trait 1 and trait 2, two independent SNPs; H4: Association with trait 1 and trait 2, one shared SNP. Genes that demonstrated a high posterior probability of hypothesis 4 (PP.H4 > 75%) indicate the disease risk and placenta gene expression were controlled by the same genetic variant; and genes that demonstrated a high posterior probability of hypothesis 3 (PP.H3 > 75%) indicate the disease risk and placenta gene expression were controlled by distinct genetic variant at the locus.

## Electronic supplementary material

Supplementary Figures and Tables

Table S4
