# Supplementary material for: Genetic Pleiotropy between Nicotine Dependence and Respiratory Outcomes
Source: Sci Rep. 2017 Dec 4;7:16907. doi: 10.1038/s41598-017-16964-4 (PMC5715160; doi:10.1038/s41598-017-16964-4)
Supplement: Supplementary file 1 — Supplementary Figures and Tables [file 41598_2017_16964_MOESM1_ESM.pdf]

Supplement for **MANUSCRIPT NUMBER**: SREP-17-42180A

Genetic Pleiotropy between Nicotine Dependence and Respiratory Outcomes

Jushan Zhang[1,2], Shouneng Peng[3,4], Haoxiang Cheng[3,4], Yoko Nomura[5,6], Antonio Fabio Di Narzo[3*], Ke Hao[1,3,4*]

[1] Department of Respiratory Medicine, Shanghai Tenth People's Hospital, Tongji University, Shanghai, China

[2] School of Life Sciences and Technology, Tongji University, Shanghai, China

[3] Department of Genetics and Genomic Sciences, Icahn School of Medicine at Mount Sinai, New York, NY, USA

[4] Icahn Institute of Genomics and Multiscale Biology, Icahn School of Medicine at Mount Sinai, New York, NY, USA

[5] Department of Psychology, Queens College & Graduate Center, the City University of New York, New York, NY, USA

[6] Department of Psychiatry, Icahn School of Medicine at Mount Sinai, New York, NY, USA

***Corresponding Author:** dinarzo.antonio@gmail.com and ke.hao@mssm.edu

Figure S1: LocusZoom plot showing the association of smoking adjusted lung function traits and known nicotine dependent locus 15q25.1.

A. 15q25.1, FEV1; Lead SNP: rs1051730

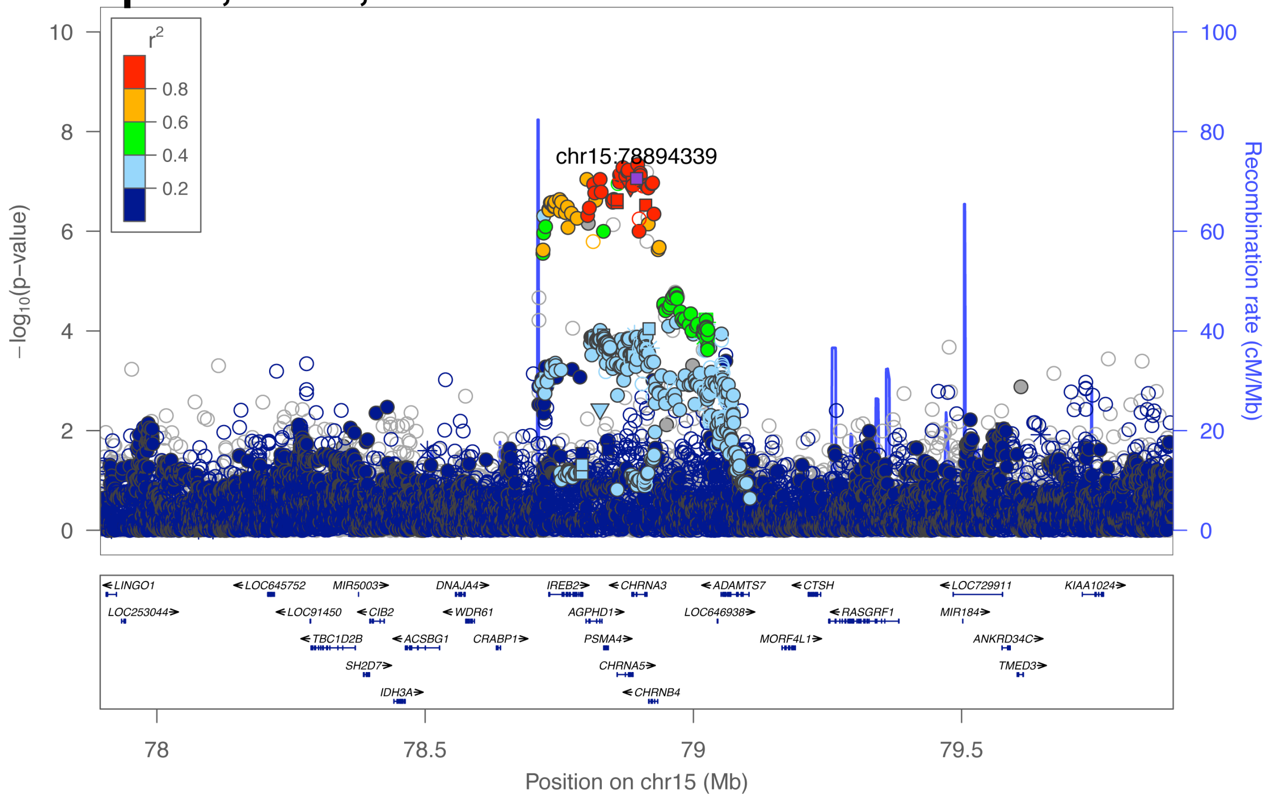

B. 15q25.1, FVC; Lead SNP: rs1051730

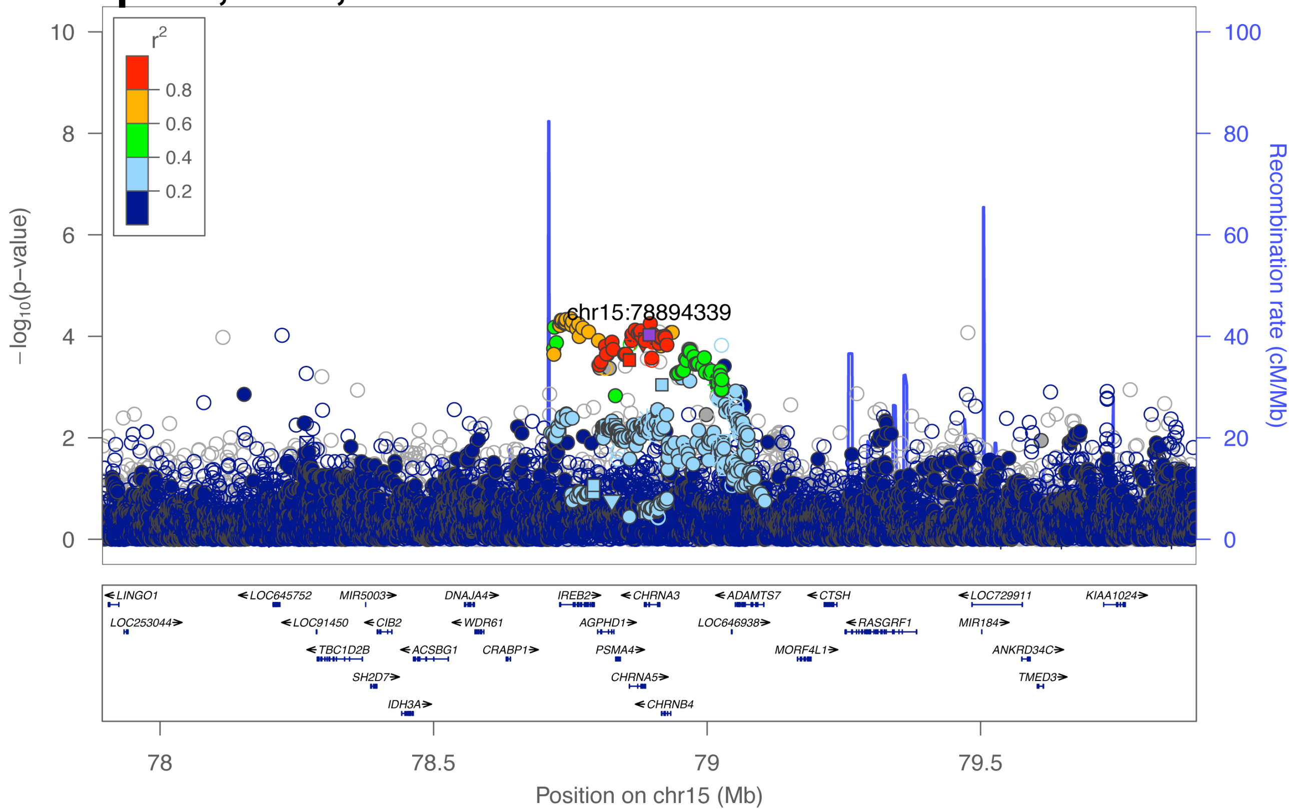

C. 15q25.1, FEV1/FVC RATIO; Lead SNP: rs1051730

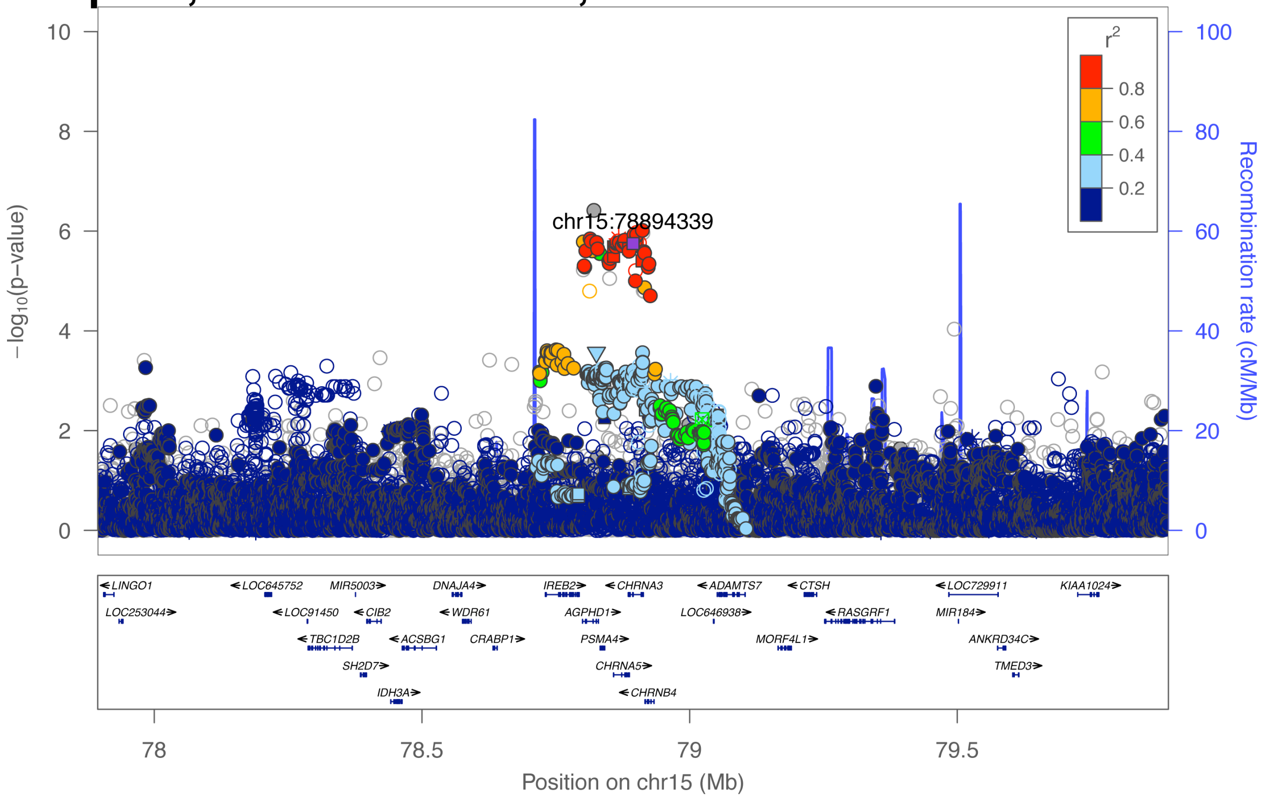

Figure S2: LocusZoom plot showing the association of smoking adjusted lung function traits and known nicotine dependent locus 10q23.32.

A. 10q23.32, FEV1; Lead SNP: rs1329650

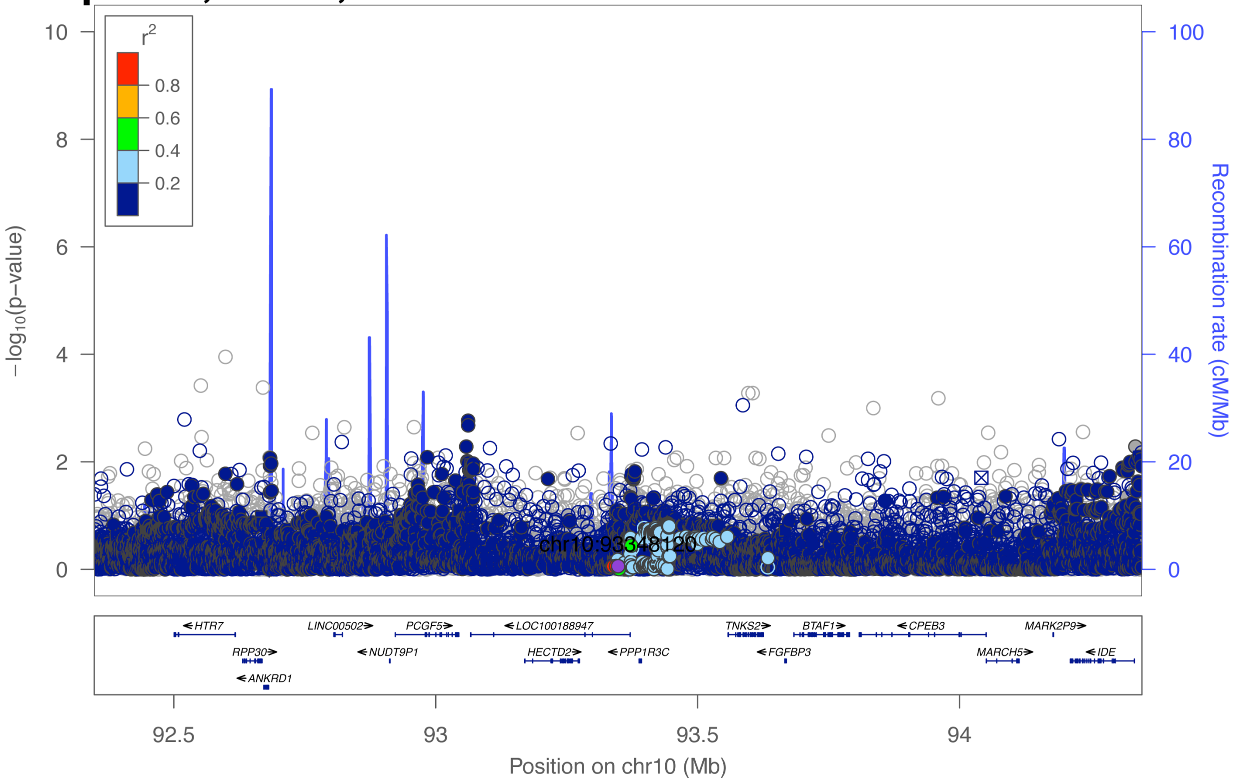

B. 10q23.32, FVC; Lead SNP: rs1329650

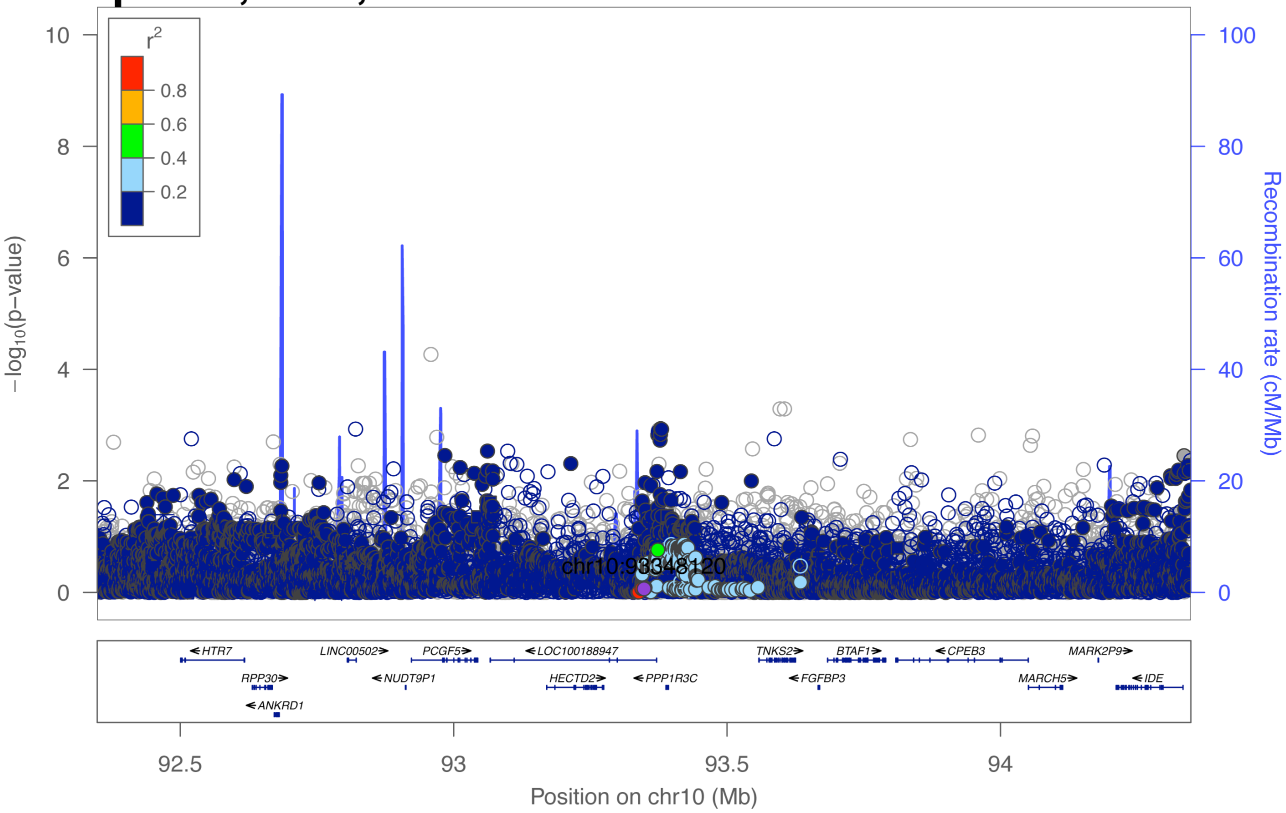

C. 10q23.32, FEV1/FVC; Lead SNP: rs1329650

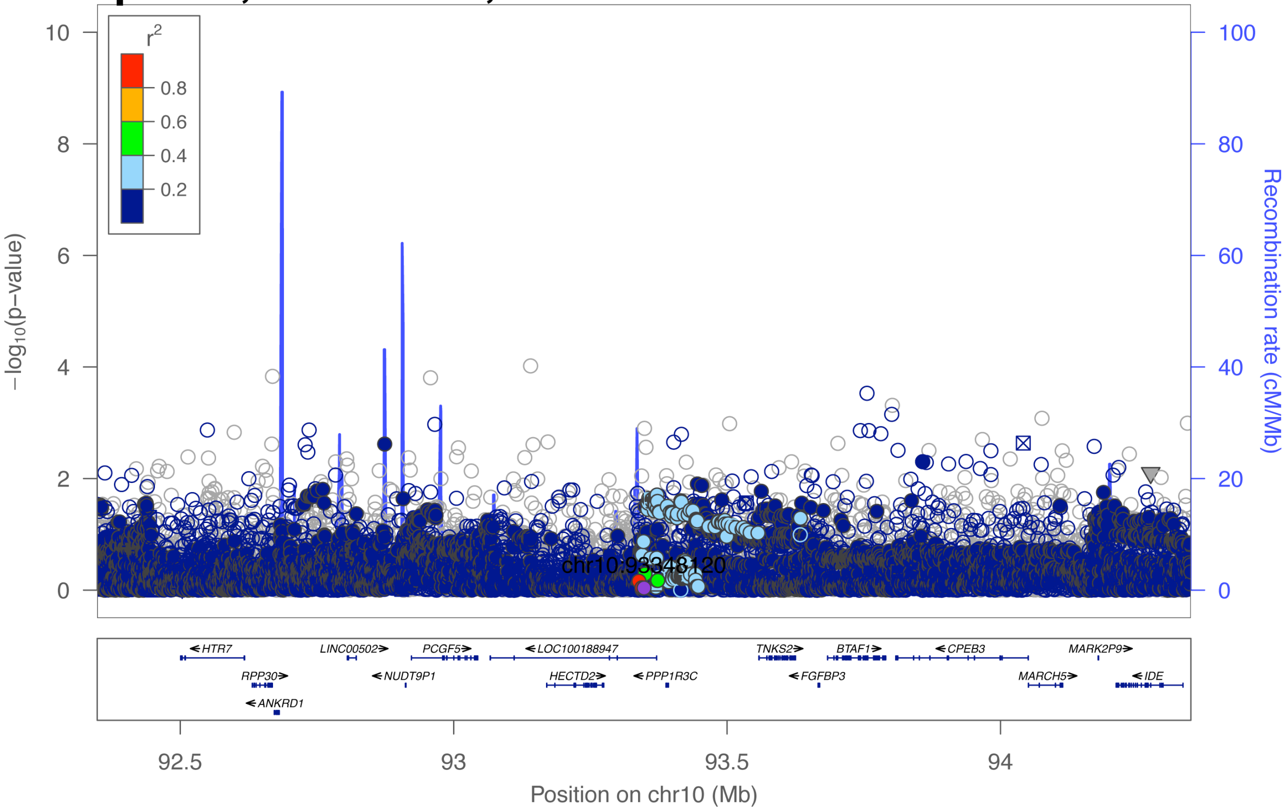

Figure S3: LocusZoom plot showing the association of smoking adjusted lung function traits and known nicotine dependent locus 8p11.21.

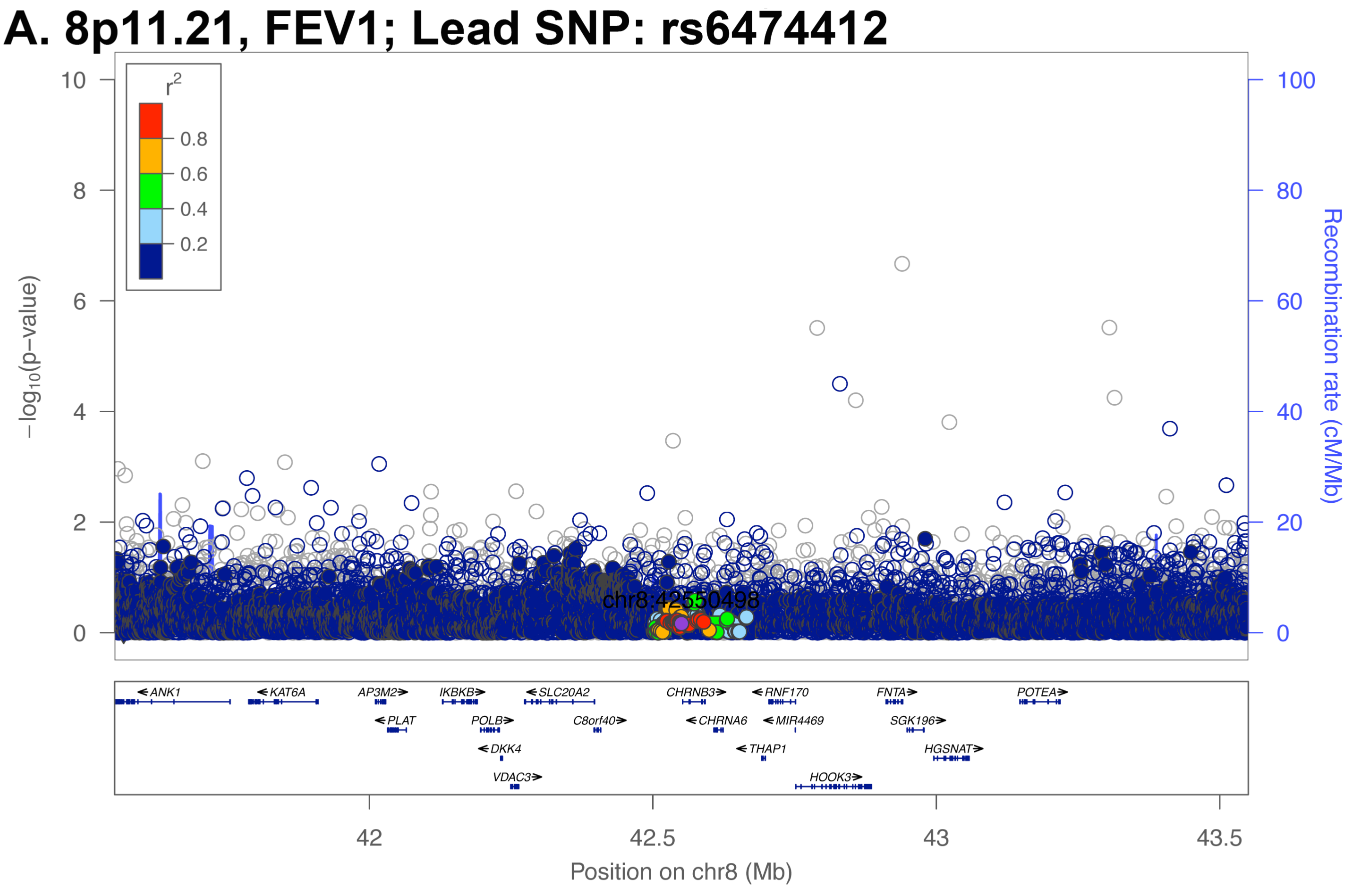

B. 8p11.21, FVC; Lead SNP: rs6474412

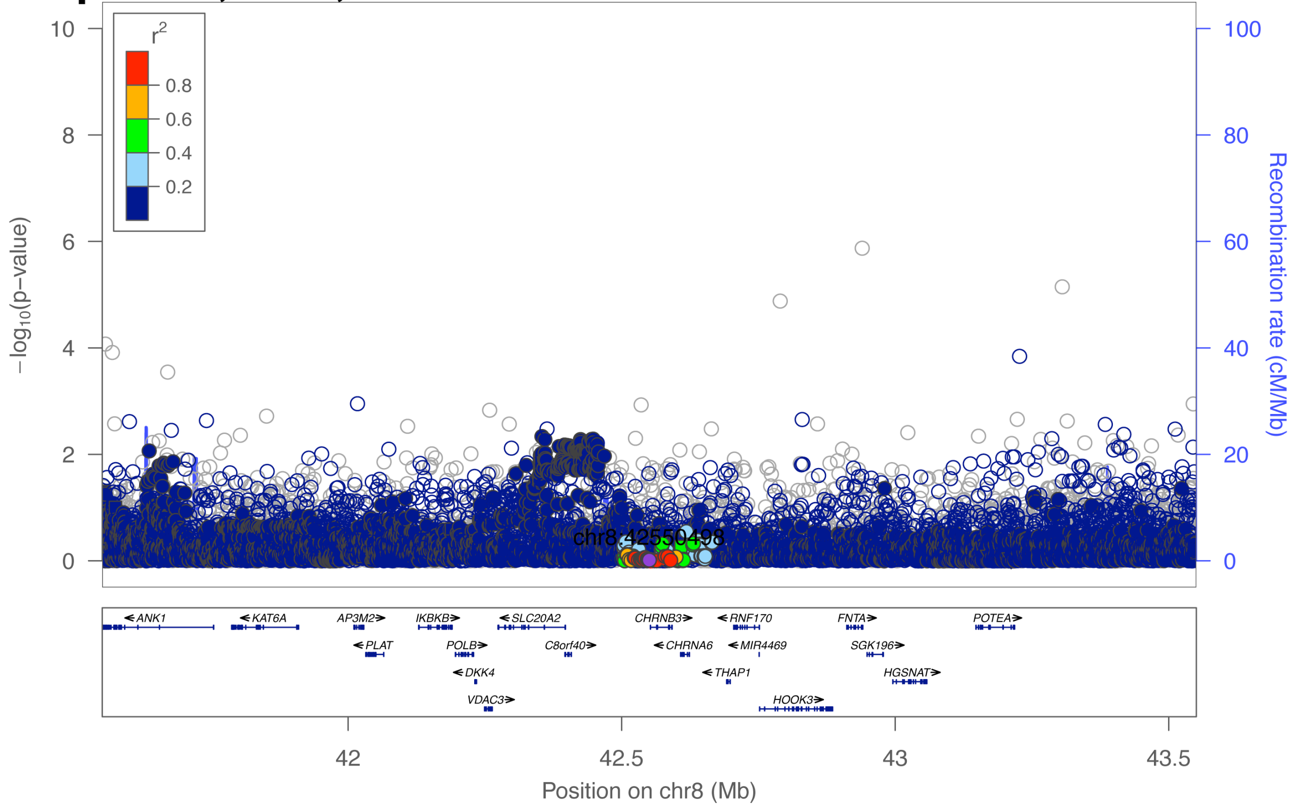

C. 8p11.21, FEV1/FVC RATIO; Lead SNP: rs6474412

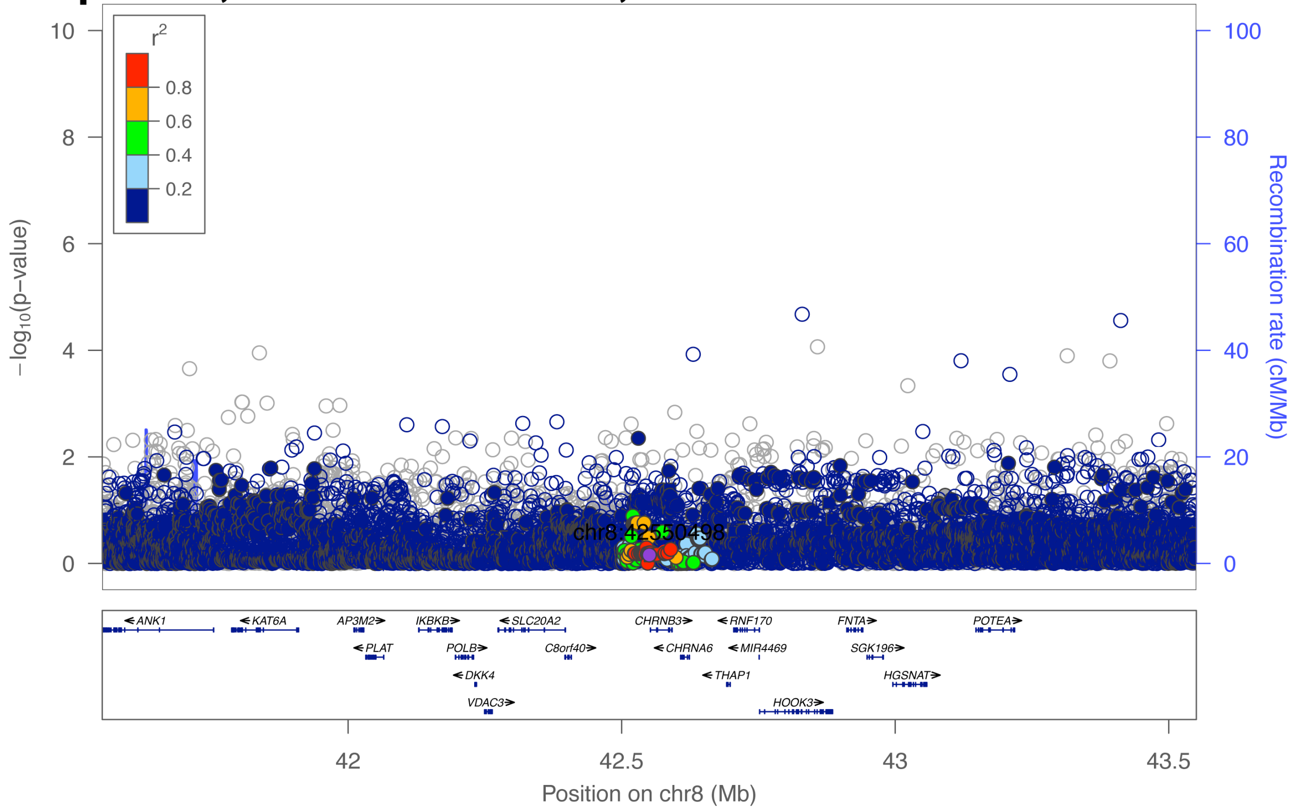

Figure S4: LocusZoom plot showing the association of smoking adjusted lung function traits and known nicotine dependent locus 19q13.2.

A. 19q13.2, FEV1; Lead SNP: rs3733829

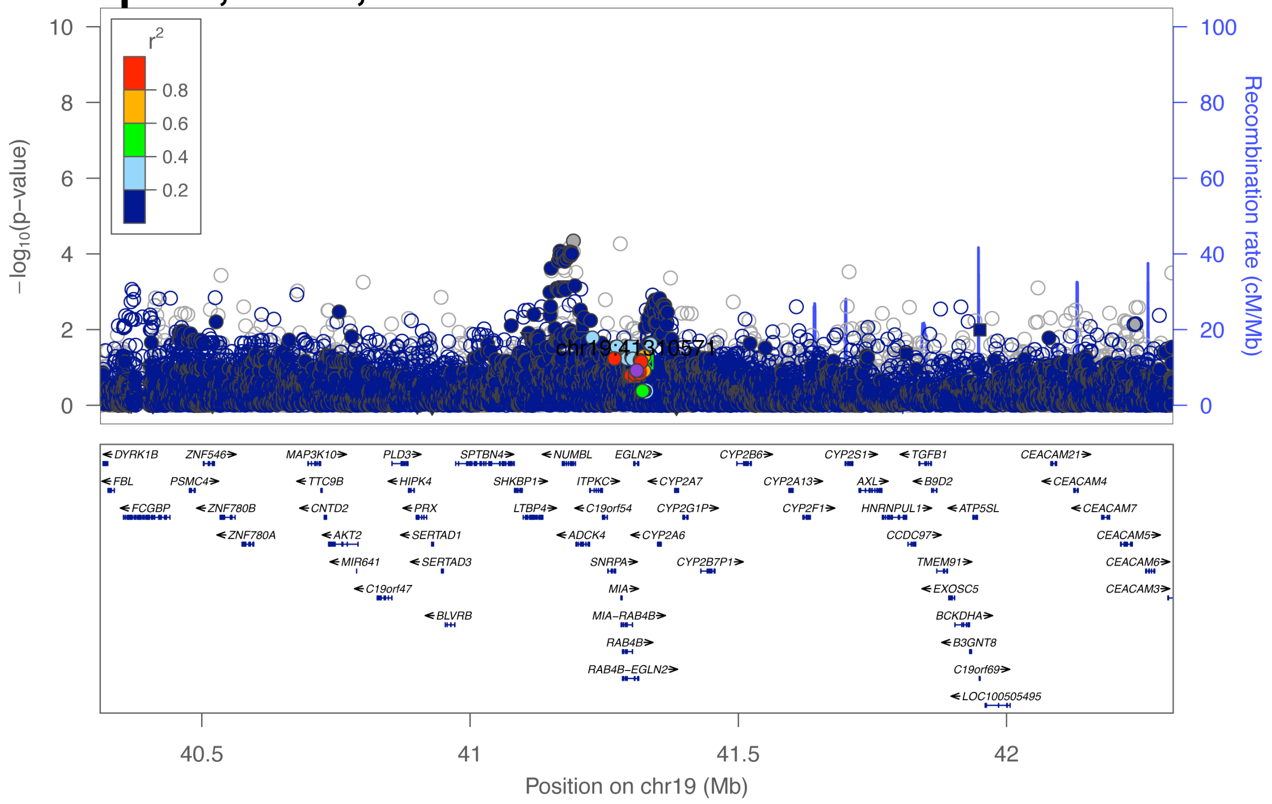

B. 19q13.2, FVC; Lead SNP: rs3733829

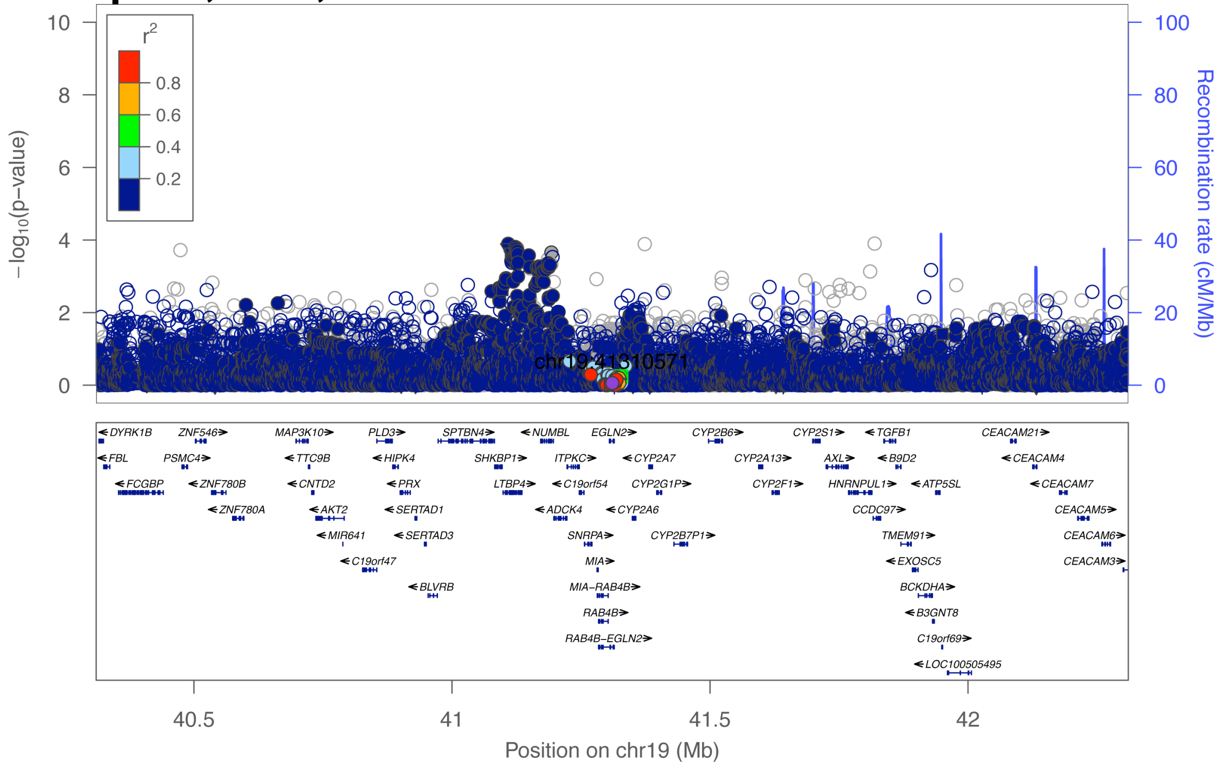

C. 19q13.2, FEV1/FVC; Lead SNP: rs3733829

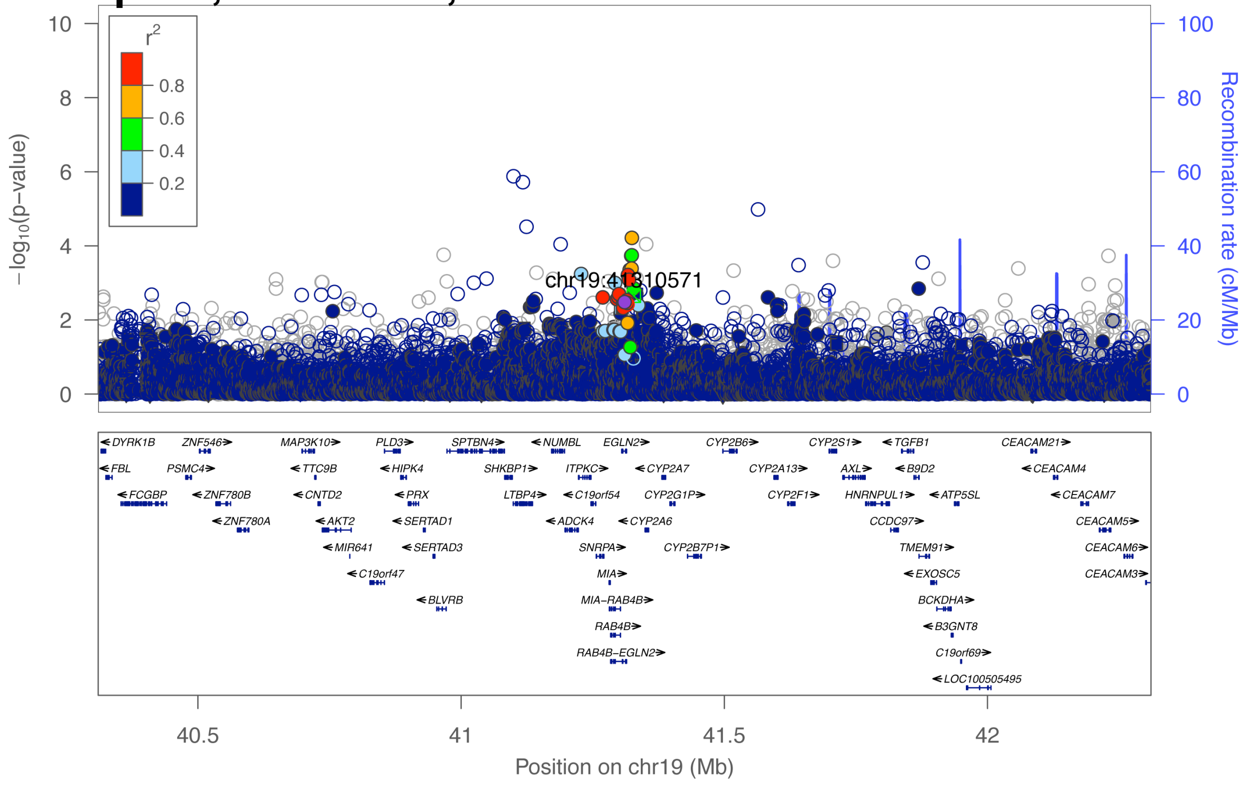

Figure S5: LocusZoom plot showing the association of smoking adjusted lung function traits and known nicotine dependent locus 11p14.1.

A. 11p14.1, FEV1; Lead SNP: rs6265

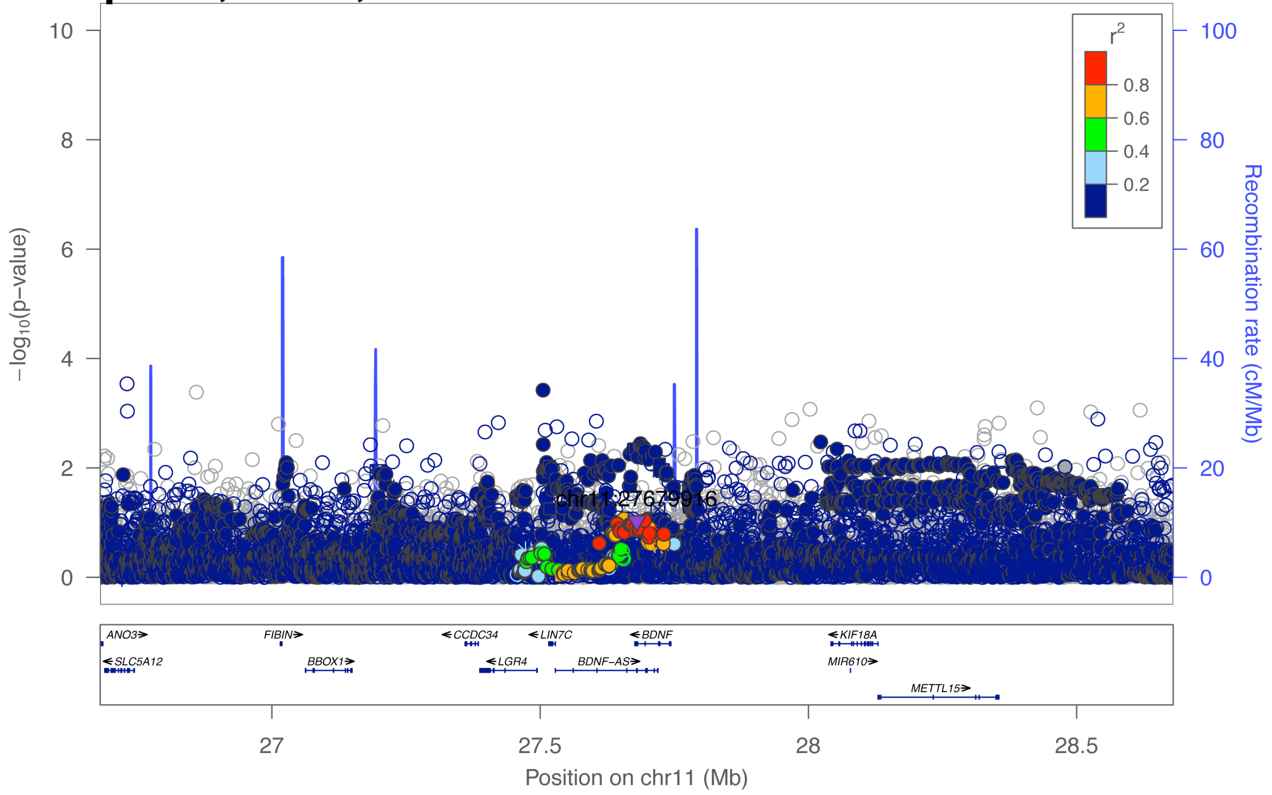

B. 11p14.1, FVC; Lead SNP: rs6265

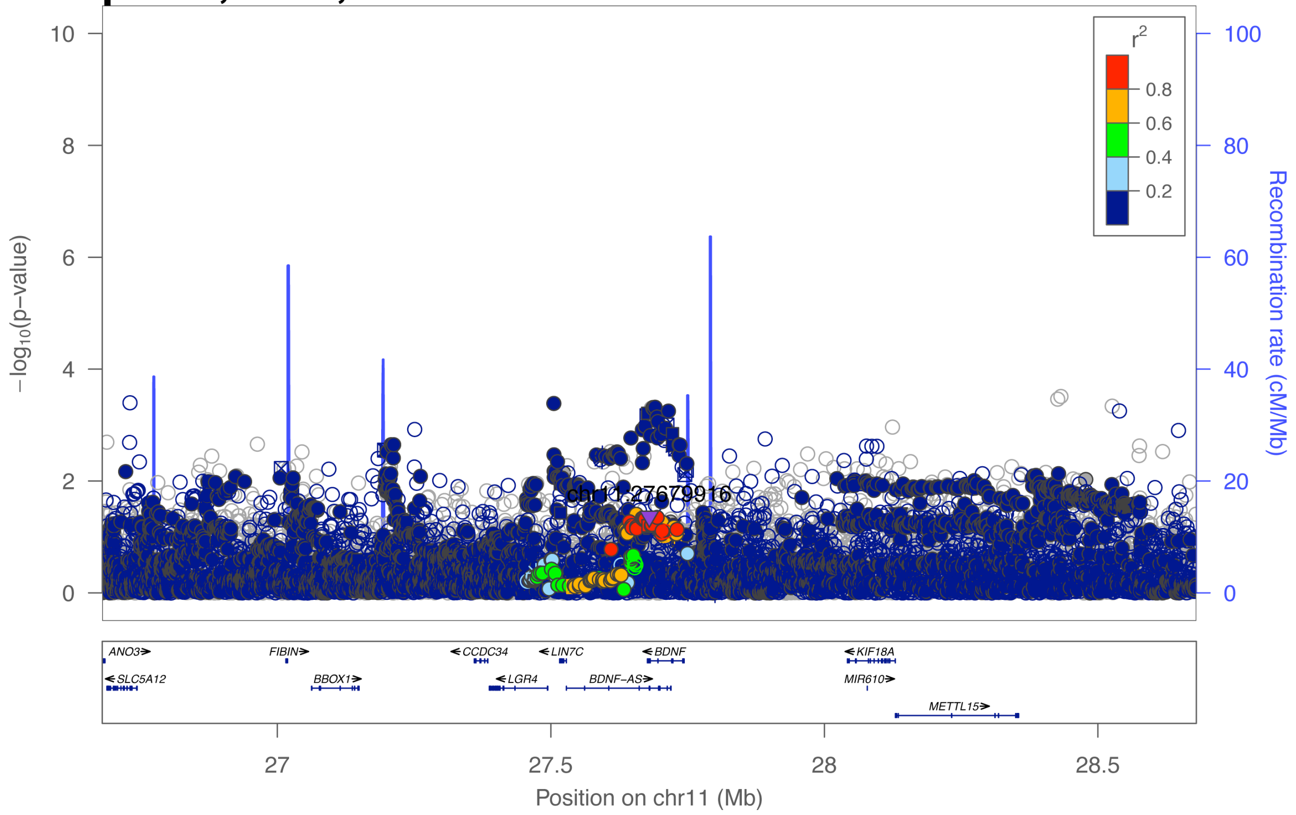

C. 11p14.1, FEV1/FVC; Lead SNP: rs6265

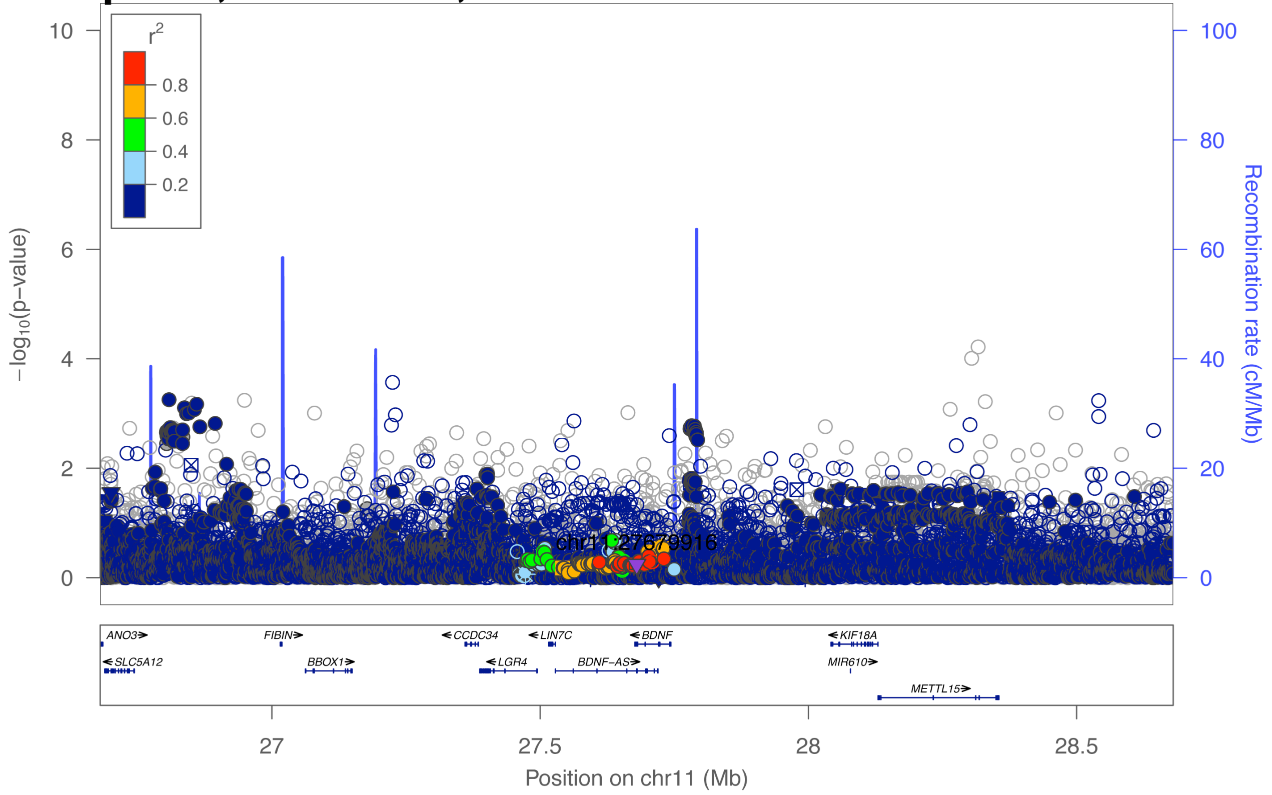

Figure S6: LocusZoom plot showing the association of smoking adjusted lung function traits and known nicotine dependent locus 9q34.2.

A. 9q34.2, FEV1; Lead SNP, rs3025343

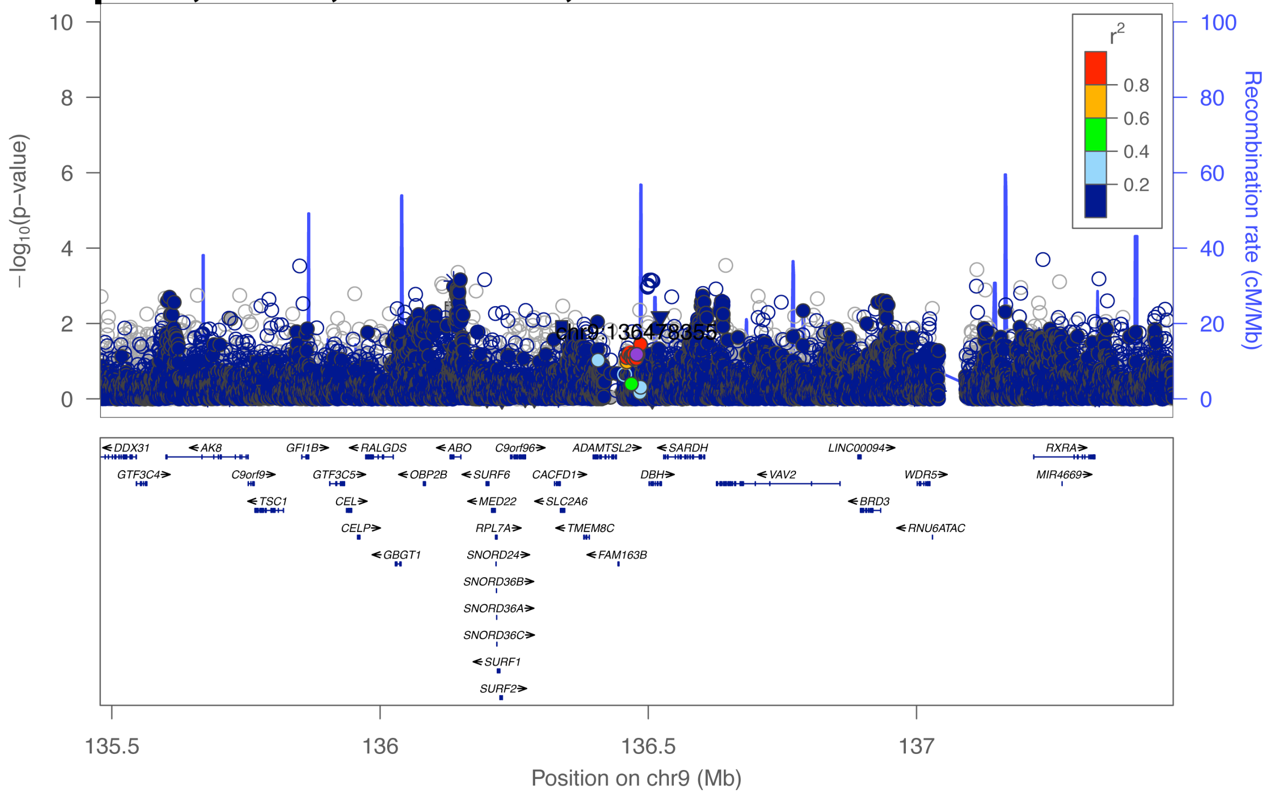

B. 9q34.2, FVC; Lead SNP, rs3025343

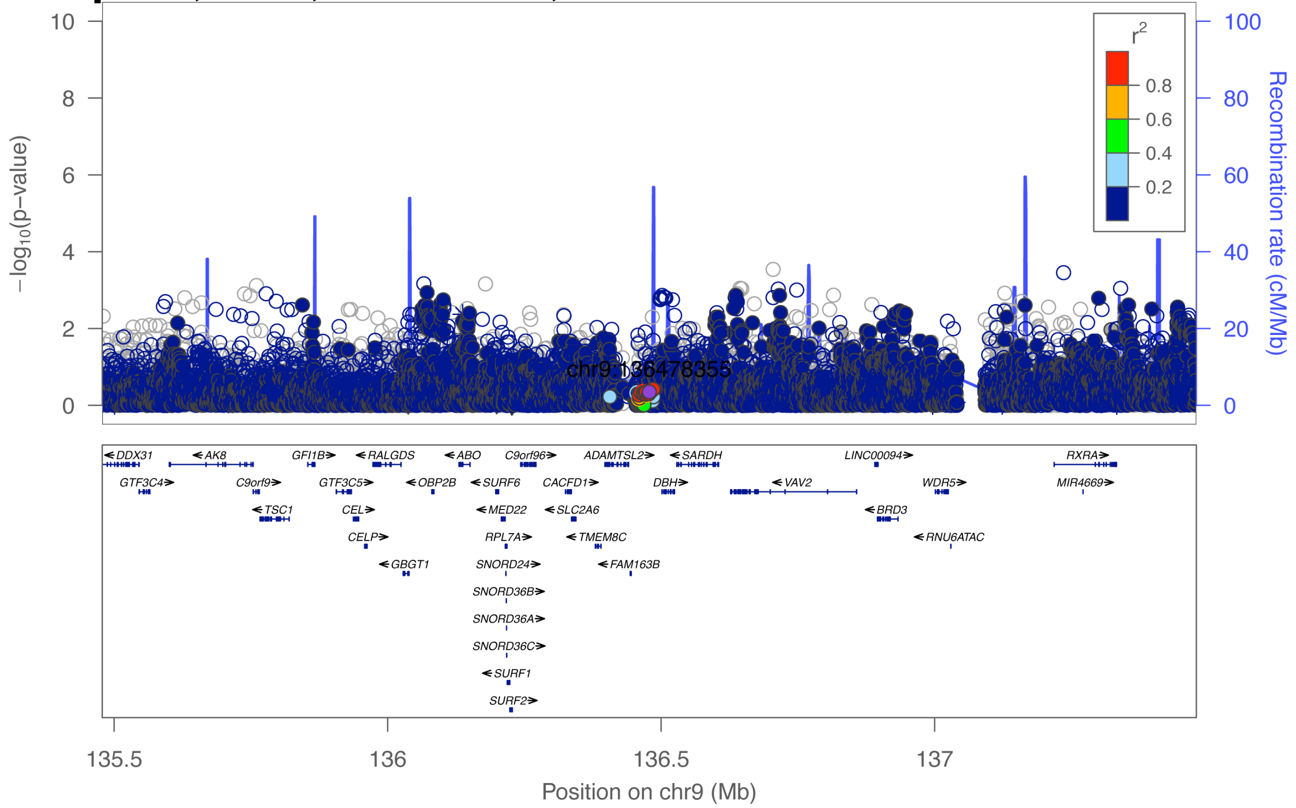

C. 9q34.2, FEV1/FVC RATIO; Lead SNP, rs3025343

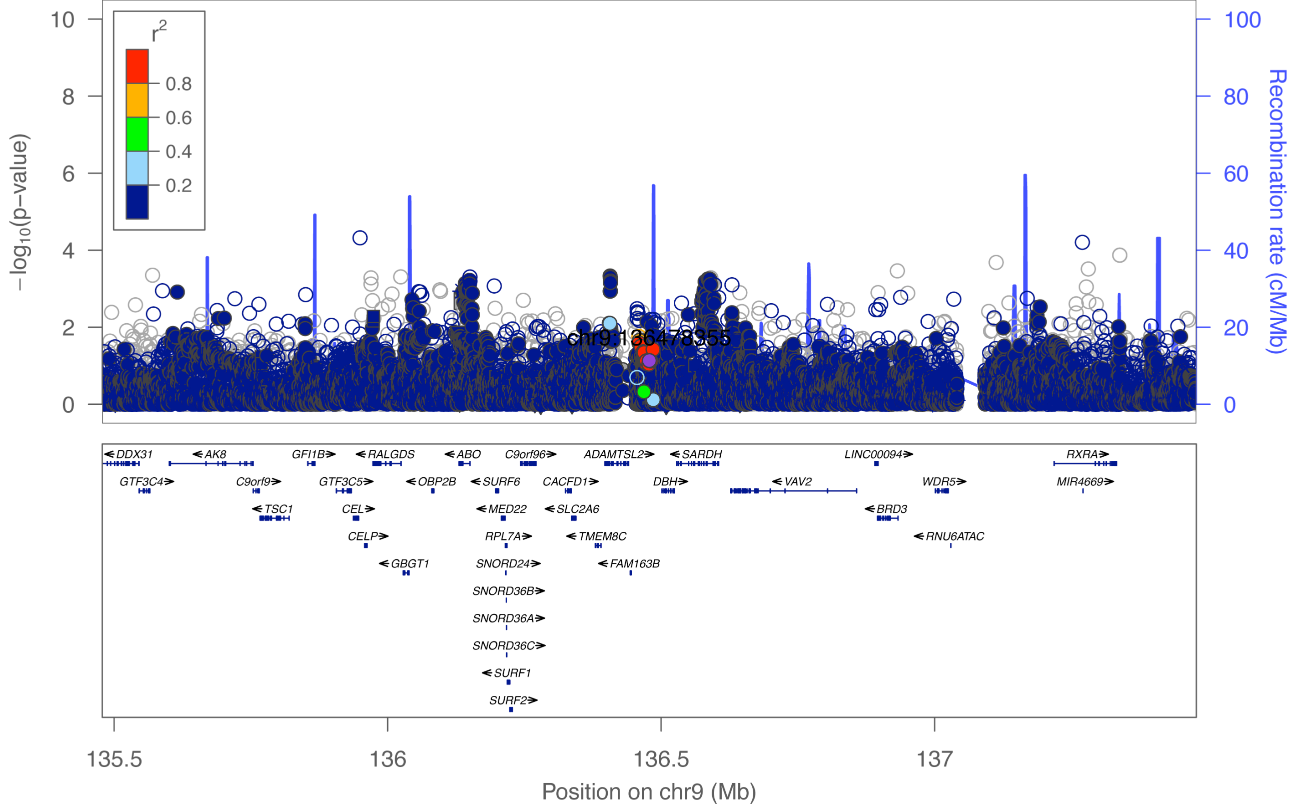

Table S1, Number of SNPs associated with Nicotine Dependence Phenotypes and Lung Function Phenotypes at various pvalue threshold

GWAS pvalue	Nicotine Dependence Phenotypes				Lung Function Phenotypes		
threshold	CPD	Evrsmk	Former	Logonset	FEV_1	FVC	FEV_1/FVC
1.0E-08	99	0	0	0	1176	900	927
1.0E-07	107	0	1	0	1519	1102	1450
1.0E-06	123	1	7	1	2143	1498	2132
1.0E-05	160	59	117	17	3182	2370	3104
1.0E-04	361	469	505	204	4977	4032	5708
1.0E-03	2791	3977	3082	2043	11716	10556	12827
1.0E-02	23835	30816	25779	22959	43953	42841	44805
1.0E-01	222330	243386	225739	216760	264660	264545	258992

CDP, cigarettes per day; Evrsmk, ever smoker; Former, former smoker.

Table S2. Overlap of GWAS signals for Ever Smoking and Lung Function.

Lung Function Traits	GWAS P value threshold	Overlap OR	Number of overlap SNPs	N of overlap SNPs of divergent risk direction	% SNPs of divergent risk direction	p-value of divergent risk direction
FEV_1	1.0E-06	-	0	0	-	-
	1.0E-05	-	0	0	-	-
	1.0E-04	-	0	0	-	-
	1.0E-03	0.57	13	9	69.23	0.313
	1.0E-02	1.40	902	393	43.57	0.000214
	1.0E-01	1.04	31971	16019	50.10	0.76
FVC	1.0E-06	-	0	0	-	-
	1.0E-05	-	0	0	-	-
	1.0E-04	-	0	0	-	-
	1.0E-03	1.63	33	11	33.33	0.10
	1.0E-02	1.26	796	438	55.03	0.007
	1.0E-01	1.06	32319	16330	50.53	0.08
FEV_1/FVC Ratio	1.0E-06	-	0	0	-	-
	1.0E-05	-	0	0	-	-
	1.0E-04	-	0	0	-	-
	1.0E-03	0.28	7	0	0	2.21E-02
	1.0E-02	1.38	904	390	43.14	7.56E-05
	1.0E-01	1.05	31583	15129	47.90	3.09E-13

Table S3. Overlap of GWAS signals between smoking logonset and lung function traits.

Lung Function Traits	GWAS P value threshold	Overlap OR	Number of overlap SNPs	N of overlap SNPs of divergent risk direction	% SNPs of divergent risk direction	p-value of divergent risk direction
FEV_1	1.0E-06	-	0	0	-	-
	1.0E-05	-	0	0	-	-
	1.0E-04	-	0	0	-	-
	1.0E-03	2.53	29	7	24.14	0.0117
	1.0E-02	1.15	556	286	51.44	0.577
	1.0E-01	1.04	28568	14036	49.13	0.00506
FVC	1.0E-06	-	0	0	-	-
	1.0E-05	-	0	0	-	-
	1.0E-04	2.52	1	0	0	1.00
	1.0E-03	4.11	42	10	23.81	1.47E-03
	1.0E-02	1.16	547	201	36.75	1.46E-09
	1.0E-01	1.03	28322	14186	50.09	8.17E-01
FEV_1/FVC Ratio	1.0E-06	-	0	0	-	-
	1.0E-05	-	0	0	-	-
	1.0E-04	-	0	0	-	-
	1.0E-03	0.24	3	3	100	0.295
	1.0E-02	1.09	543	310	57.09	0.00168
	1.0E-01	1.02	27452	13785	50.21	0.545

Table S5. Overlap of GWAS signals nicotine dependence (CDP) and lung function (LF_{adj}) stratified by allele direction

**LF_{adj} trait**	**Pvalue threshold**	**Divergent direction allele**		**Consistent direction allele**	
		N. overlap SNP	Enrichment Fold	N. overlap SNP	Enrichment Fold
FEV_1	1.0E-06	26	277	0	-
	1.0E-05	26	141	0	-
	1.0E-04	42	76.1	0	-
	1.0E-03	65	6.94	6	0.97
	1.0E-02	451	1.72	375	1.61
FVC	1.0E-06	0	-	0	-
	1.0E-05	0	-	0	-
	1.0E-04	13	26.8	0	-
	1.0E-03	47	5.61	2	0.35
	1.0E-02	491	1.92	378	1.68
FEV_1/FVC Ratio	1.0E-06	0	-	0	-
	1.0E-05	14	83.4	0	-
	1.0E-04	14	22.0	0	-
	1.0E-03	39	4.62	0	-
	1.0E-02	407	1.65	262	0.99

Pvalue threshold, GWAS pvalue threshold applied to both TAG GWAS (CPD phenotype) and UKBB lung function GWAS (FEV_1, FVC, FEV_1/FVC Ratio phenotype). Divergent direction allele, the SNPs where the different allele association with higher cigarettes per day and higher FEV_1, FVC, FEV_1/FVC Ratio; Consistent direction allele, the SNPs where the same allele association with higher cigarettes per day and higher FEV_1, FVC, FEV_1/FVC Ratio.

Table S6. Overlap of GWAS signals nicotine dependence (FORMER) and lung function (LF_{adj}) stratified by allele direction

**LF_{adj} trait**	**Pvalue threshold**	**Divergent direction allele**		**Consistent direction allele**	
		N. overlap SNP	Enrichment Fold	N. overlap SNP	Enrichment Fold
FEV_1	1.0E-06	0	-	0	-
	1.0E-05	0	-	4	42.1
	1.0E-04	0	-	20	32.2
	1.0E-03	21	3.03	172	18.4
	1.0E-02	290	1.21	912	3.19
FVC	1.0E-06	0	-	0	-
	1.0E-05	0	-	0	-
	1.0E-04	0	-	8	11.6
	1.0E-03	21	3.35	123	14.1
	1.0E-02	336	1.49	763	2.63
FEV_1/FVC Ratio	1.0E-06	0	-	0	-
	1.0E-05	0	-	4	28.6
	1.0E-04	0	-	23	33.0
	1.0E-03	3	0.337	165	18.5
	1.0E-02	343	1.28	723	2.66

Pvalue threshold, GWAS pvalue threshold applied to both TAG GWAS (FORMER phenotype) and UKBB lung function GWAS (FEV_1, FVC, FEV_1/FVC Ratio phenotype). Divergent direction allele, the SNPs where the different allele association with higher current vs former smoker and higher FEV_1, FVC, FEV_1/FVC Ratio; Consistent direction allele, the SNPs where the same allele association with higher current vs former smoker and higher FEV_1, FVC, FEV_1/FVC Ratio.

Table S7. Overlap of GWAS signals nicotine dependence (EVRSMK) and lung function (LF_{adj}) stratified by allele direction

**LF_{adj} trait**	**Pvalue threshold**	**Divergent direction allele**		**Consistent direction allele**	
		N. overlap SNP	Enrichment Fold	N. overlap SNP	Enrichment Fold
FEV_1	1.0E-06	0	-	0	-
	1.0E-05	0	-	0	-
	1.0E-04	0	-	0	-
	1.0E-03	9	0.711	4	0.399
	1.0E-02	393	1.15	509	1.69
FVC	1.0E-06	0	-	0	-
	1.0E-05	0	-	0	-
	1.0E-04	0	-	0	-
	1.0E-03	11	0.993	22	2.41
	1.0E-02	438	1.37	358	1.15
FEV_1/FVC Ratio	1.0E-06	0	-	0	-
	1.0E-05	0	-	0	-
	1.0E-04	0	-	0	-
	1.0E-03	0	-	7	0.597
	1.0E-02	390	1.21	514	1.53

Pvalue threshold, GWAS pvalue threshold applied to both TAG GWAS (EVRSMK phenotype) and UKBB lung function GWAS (FEV_1, FVC, FEV_1/FVC Ratio phenotype). Divergent direction allele, the SNPs where the different allele association with higher ever vs never smoked and higher FEV_1, FVC, FEV_1/FVC Ratio; Consistent direction allele, the SNPs where the same allele association with higher ever vs never smoked and higher FEV_1, FVC, FEV_1/FVC Ratio.

Table S8. Overlap of GWAS signals nicotine dependence (LOGONSET) and lung function (LF_{adj}) stratified by allele direction

**LF_{adj} trait**	**Pvalue threshold**	**Divergent direction allele**		**Consistent direction allele**	
		N. overlap SNP	Enrichment Fold	N. overlap SNP	Enrichment Fold
FEV_1	1.0E-06	0	-	0	-
	1.0E-05	0	-	0	-
	1.0E-04	0	-	0	-
	1.0E-03	7	1.14	22	4.14
	1.0E-02	286	1.15	270	1.14
FVC	1.0E-06	0	-	0	-
	1.0E-05	0	-	0	-
	1.0E-04	0	-	1	4.24
	1.0E-03	10	1.91	32	6.40
	1.0E-02	201	0.846	346	1.48
FEV_1/FVC Ratio	1.0E-06	0	-	0	-
	1.0E-05	0	-	0	-
	1.0E-04	0	-	0	-
	1.0E-03	3	0.410	0	-
	1.0E-02	310	1.21	233	0.977

Pvalue threshold, GWAS pvalue threshold applied to both TAG GWAS (LOGONSET phenotype) and UKBB lung function GWAS (FEV_1, FVC, FEV_1/FVC Ratio phenotype). Divergent direction allele, the SNPs where the different allele association with higher log age-onset smoking and higher FEV_1, FVC, FEV_1/FVC Ratio; Consistent direction allele, the SNPs where the same allele association with higher log age-onset smoking and higher FEV_1, FVC, FEV_1/FVC Ratio.
